# Postnatal care after gestational diabetes – a systematic review of clinical practice guidelines

**DOI:** 10.1186/s12884-024-06899-w

**Published:** 2024-11-04

**Authors:** Phyllis Ohene-Agyei, Ariba Iqbal, Jane E. Harding, Caroline A. Crowther, Luling Lin

**Affiliations:** Liggins Institute, University of Auckland, 85 Park Road, Grafton, Auckland, 1023 New Zealand

**Keywords:** Gestational diabetes mellitus, Pregnancy-induced diabetes, Hyperglycaemia, Glucose intolerance, Postpartum care, Practice guidelines, Evidence gaps

## Abstract

**Background:**

Gestational diabetes mellitus (GDM) is the most common metabolic disorder in pregnancy and later is associated with an increased risk of type 2 diabetes and other metabolic disorders. Consistent and evidence based postnatal care is key to improving maternal long-term health. We therefore aimed to review and compare recommendations of national and international clinical practice guidelines (CPG) for postnatal care after GDM and identify any evidence gaps in recommendations needing further research.

**Methods:**

We searched five databases and forty professional organization websites for CPGs providing recommendations for postnatal care after GDM. CPGs which had full versions in English, endorsed, prepared, or authorized by a professional body, and published between 2013 and 2023 were eligible for inclusion. Two reviewers independently screened the articles, extracted the recommendations, and appraised the included CPGs using the Appraisal of Guidelines, Research, and Evaluation (AGREE) II tool.

**Results:**

Twenty-six CPGs from 22 countries were included. Twelve CPGs (46%) were appraised as low quality with the lowest scoring domains being rigor of development and editorial independence. We found little high certainty evidence for most recommendations and few recommendations were made for maternal mental health and postpartum metabolic screening. Evidence gaps pertained to postpartum glucose screening, including frequency, tests, and ways to improve uptake, evaluation of effective uptake of lifestyle interventions, and ongoing long-term follow up care.

**Conclusions:**

Most of the postnatal care recommendations in GDM guidelines are not based on high certainty evidence. Further efforts are needed to improve the global evidence base for postnatal care after GDM to improve long-term maternal health.

**Protocol Registration:**

This review was registered in PROSEPRO (CRD42023454900).

**Supplementary Information:**

The online version contains supplementary material available at 10.1186/s12884-024-06899-w.

## Background

Gestational diabetes mellitus (GDM) is defined as glucose intolerance with its first onset in pregnancy which does not meet the threshold of overt diabetes [1, 2]. It is the most common metabolic disorder in pregnancy, complicating an estimated one in seven pregnancies globally [3], and can result in significant adverse short and long-term complications for mother and child. Women diagnosed with GDM are at an increased risk for complications such as pre-eclampsia [4], induction of labor [4, 5], caesarean delivery [5, 6], a large for gestational age baby [5, 6], and depression [7]. In the long-term these women also have an increased risk of recurrence of GDM in subsequent pregnancies, and of developing impaired glucose metabolism, type 2 diabetes, and cardiovascular diseases [8–10]. A recent meta-analysis of twenty observational studies with low risk of bias, estimated the risk of progression to type 2 diabetes as almost ten-fold higher in women with GDM compared to women without (RR: 9.51, 95% CI 7.14 to 12.67) [11]. Early detection of dysglycaemia by postpartum glucose screening, followed by appropriate management, is encouraged to address this risk. Dietary and lifestyle advice to optimize postpartum weight, glucose surveillance, appropriate family planning services, and long-term follow-up have also been highlighted as domains of postnatal care after GDM that can reduce long-term risks [12, 13]. However, reports generally show a low uptake of postnatal care, especially postpartum glucose screening, partly due to disparities in recommendations by professional bodies on screening schedules, tests, and frequencies [14, 15], and lack of consensus among health professionals [16]. Additionally, some studies have reported that compared to their antenatal care, most women felt that they were not adequately supported by health professionals during the postnatal period [17, 18].

Clinical practice guidelines (CPGs) offer structure and guidance in health care delivery by recommending optimum care. GPGs improve care by promoting interventions of proven benefit and discouraging ineffective or potentially harmful interventions [19, 20]. A systematic review on quality of CPGs over the last two decades reported a marked improvement in the clarity and scope of CPGs but also noted that most guidelines were not adequately applicable to everyday clinical practice [20]. A content appraisal of fourteen GDM guidelines noted that most recommendations were focused on antenatal care [21]. Considering the significant long-term risks following GDM and the increasing prevalence of GDM globally, recommendations for postpartum glucose screening and follow-up care after GDM need to be prioritized. We aimed to assess national and international clinical practice guideline recommendations for postnatal care and later follow-up after GDM. Specifically, we sought to compare national and international guideline recommendations on several postnatal care domains including postpartum glucose screening, lifestyle advice, breastfeeding, family planning, and long-term follow up. We also examined the evidence base and certainty of these recommendations and identified the gaps in recommendations that may need further research. Lastly, we compared guideline recommendations between regions (high income versus low-and-middle income countries).

## Methods

### Protocol and registration

This review was registered in PROSEPRO (CRD42023454900) and reported in accordance with the Preferred Reporting Items for Systematic Reviews and Meta-Analyses (PRISMA) guidelines (Table S1) [22].

### Eligibility criteria

CPGs meeting the following criteria were included: (1) full guideline or statement available in English; (2) developed, authorised or endorsed by a nationally or internationally recognized committee or body; and (3) provide recommendations for postnatal care after GDM related to postpartum glucose screening, lifestyle advice related to future metabolic risks, breastfeeding, family planning/ contraception, and long-term follow up; and (4) published or updated in the last 10 years (since 2013). When more than one version of a CPG was found, only the most recent version was included. CPGs were excluded if they were under review, unpublished, full text not available, or developed for use in one institution, such as a hospital.

### Search strategy and data sources

We used a comprehensive search strategy between 21st August and 18th September 2023 using medical subject headings and keywords related to “gestational diabetes”, “clinical practice guidelines”, “postnatal”, and “recommendations” to search Ovid MEDLINE, EMBASE, CINAHL Complete, Guideline International Network, and National Institute for Health and Clinical Excellence (NICE) (Table S2). Forty organizational websites, including those of professional societies and committees, and the World Health Organization (WHO) were also searched. Additionally, the reference lists of studies were hand searched.

### Study selection

Records were uploaded to Covidence [23] and independently screened by two reviewers (PO and AI) based on the title and abstract and then full text, and the reasons for exclusions documented. Disagreements were resolved by discussion between the two reviewers.

### Data extraction

Data from eligible CPGs were independently extracted by two researchers (PO and AI) into a data record form. The data extracted included CPG characteristics (title, year of publication/ update, affiliated professional organization, and economy region), recommendations, evidence base behind recommendations (strength of recommendation and certainty of evidence), tools used in appraising the evidence, and evidence gaps in the recommendations. If the strength of recommendation was not reported, the wording of the recommendation was used [24]. Words such as “should” or “strongly recommend” were interpreted as a strong recommendation, and “suggest” or “consider” as weak. The Grading of Recommendations Assessment, Development and Evaluation (GRADE) tool [24] was used for assessing the certainty of evidence for recommendations. For CPGs that did not use this approach we converted their evidence grading tool to the GRADE approach (Table S3 and S4). Research gaps were extracted from the CPG when these were identified or assigned as a gap for further research, or when the evidence base of the recommendation was of low or very low certainty or expert opinion without an evidence base.

### Quality appraisal of CPGs

The quality of the GPGs was assessed independently by two reviewers (PO and AI) using the AGREE II tool [25], a 23-item tool for appraisal of quality of guidelines within six domains: scope and purpose, stakeholder involvement, rigor of development, clarity of presentation, applicability, and editorial independence. Each item was scored on a 7-point scale from 1 = strongly disagree to 7 = strongly agree, and the average scores and percentages between the two reviewers were recorded. Two global rating scores which appraise the quality of the whole guideline were also recorded. Priority was given to domains 3 (rigor of development) and 5 (applicability) to deem a GPG as high quality as these two domains have been shown to have the strongest influence on overall guideline quality [26]. The ‘rigor of development’ domain assesses the evidence base and recommendations development process, while the ‘applicability’ domain assesses strategies to improve uptake and resource implications of applying the guideline. A guideline was appraised as high quality if at least 4 domains scored ≥ 70% including the rigor of development and applicability domains, moderate quality if ≥ 50% and < 70%, and low quality if < 50%. For the global rating items, guidelines with an overall quality score of 1–2 were not recommended for use, 3–5 were recommended for use with modifications, and 6–7 recommended for use without modifications [27].

## Results

**Fig. 1:**
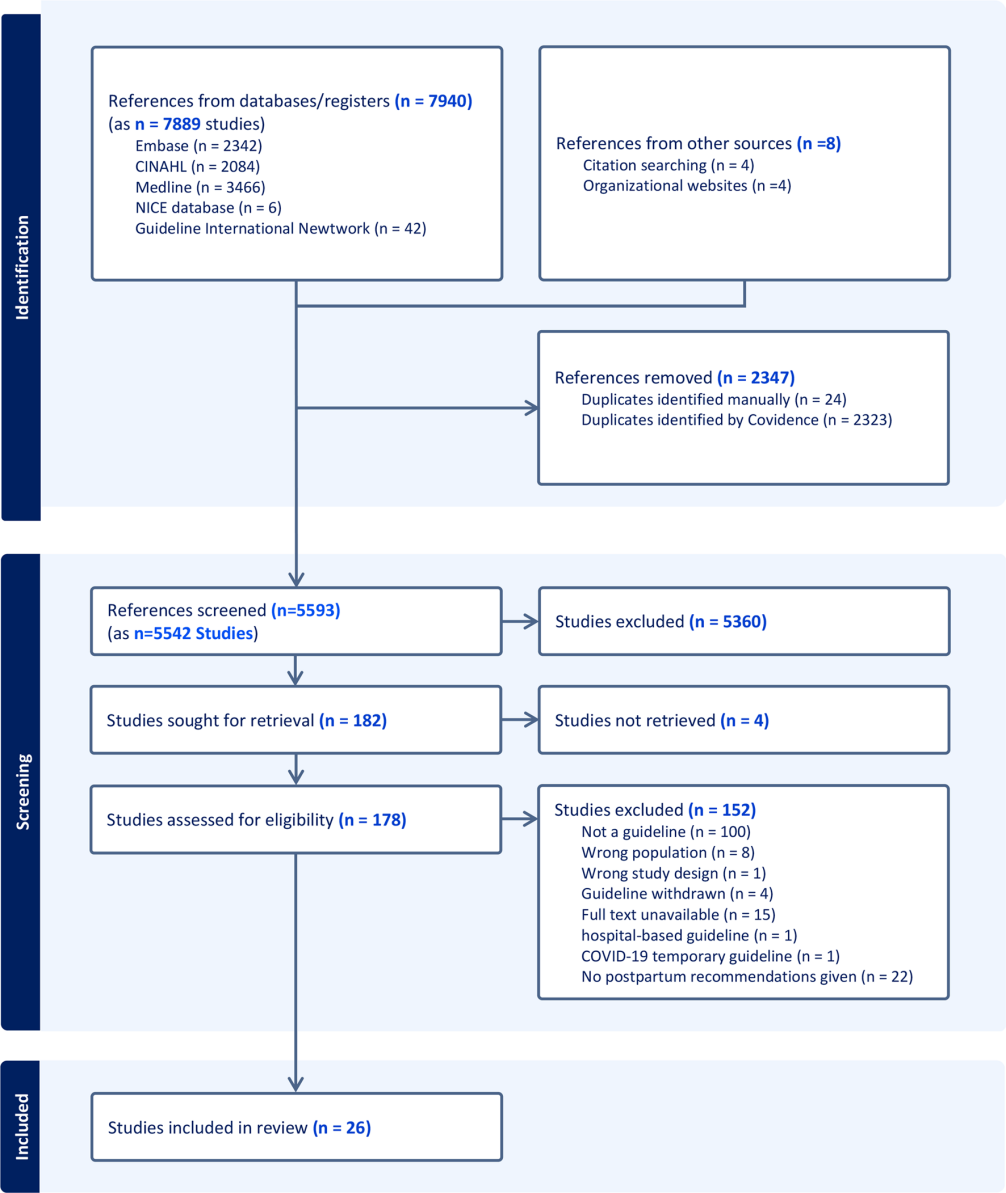
Preferred Reporting Items for Systematic Reviews and Meta-analysis (PRISMA) flow diagram outlining identification and selection of guidelines for review

The search strategy identified 7889 records from the five databases [EMBASE (*n* = 2342), CINAHL (*n* = 2084), MEDLINE (*n* = 3466), NICE database (*n* = 6), Guideline International Network (*n* = 42)] and 8 from organisational websites and citation searching. After removal of duplicates (*n* = 2347), 5542 records underwent title and abstract screening and 5360 were excluded,178 full text records were screened, and 26 CPGs were included in the systematic review (Fig. 1).

Twenty-five of the 26 included CPGs were national guidelines, and one had an international focus. The national CPGs were from 22 countries with 10 from low-and-middle income countries and 15 from high-income countries (Table 1).

**Table 1:** Characteristics of included guidelines

	Guideline title	Affiliated professional organization	Year of Publication	Country	Economy -World Bank classification
1	Management of Diabetes in Pregnancy: Standards of Care in Diabetes [28]	American Diabetes Association (ADA)	2023	USA	High Income
2	Gestational Diabetes Mellitus (GDM) Diagnosis, Therapy and Follow-Up Care [29]	German Diabetes Association (DDG) & German Association for Gynaecology and Obstetrics (DGGG)	2021	Germany	High Income
3	National Clinical Guideline: The Diagnosis and Management of Diabetes in Pregnancy [30]	Ministry of Public Health Qatar	2021	Qatar	High Income
4	Management of type 2 diabetes: A handbook for general practice [31]	Royal Australian College of General Practitioners	2021	Australia	High Income
5	Diabetes in pregnancy: management from preconception to the postnatal period [32]	National Institute for Health and Care Excellence	2015 (updated 2020)	England	High Income
6	Clinical practice guidelines on diabetes mellitus and pregnancy ΙI: Gestational diabetes mellitus [33]	Hellenic Endocrine Society & Hellenic Society of Maternal-Fetal Medicine.	2020	Greece	High Income
7	Guidelines for Screening, Diagnosis, and Management of Gestational Diabetes Mellitus [34]	Iranian Endocrine Society	2020	Islamic Republic of Iran	Lower-middle-income
8	Clinical Practice Guideline for Diagnosis, Treatment and Follow-up of Diabetes Mellitus and Its Complications [35]	The Society of Endocrinology and Metabolism of Turkey (SEMT)	2019	Türkiye	Upper-middle-income
9	Diagnosis & Management of Gestational Diabetes Mellitus: Technical and Operational Guidelines [36]	Diabetes in Pregnancy Society India	2018	India	Lower-middle-income
10	Clinical Practice Guidelines on Diabetes Mellitus in Pregnancy [37]	Philippines Obstetrical and Gynaecological Society	2018	Philippines	Lower-middle-income
11	GDM: SAFES Recommendation and Action Plan [38]	South Asian Federation of Endocrine Societies (SAFES)	2018	Bangladesh; India; Nepal; Pakistan; Sri Lanka	Lower-middle-income
12	Standards of Polish Society of Gynecologists and Obstetricians in management of women with diabetes [39]	Polish Society of Gynecologists and Obstetricians	2018	Poland	High Income
13	ACOG Practice Bulletin No. 180: Gestational Diabetes Mellitus [40]	American College of Obstetricians and Gynecologists	2017	USA	High Income
14	2018 Clinical Practice Guidelines: Diabetes and Pregnancy [41]	Diabetes Canada	2018	Canada	High Income
15	Management of diabetes: A national clinical guideline [42]	Scottish Intercollegiate Guidelines Network (SIGN)	2010 (updated 2017)	Scotland	High Income
16	SEMDSA 2017 Guidelines for the Management of Type 2 diabetes mellitus [43]	Society for Endocrinology, Metabolism and Diabetes of South Africa	2017	South Africa	Upper-middle-income
17	Management of Diabetes in Pregnancy [44]	Malaysia Health Technology Assessment Section	2017	Malaysia	Upper-middle-income
18	HKCOG Guidelines for the Management of Gestational Diabetes Mellitus [45]	The Hong Kong College of Obstetricians and Gynaecologists	2016	Hong Kong	High Income
19	Diabetes Mellitus Management Guidelines [46]	Ministry of Health, Sultanate of Oman	2015	Oman	High Income
20	FIGO Initiative on Gestational Diabetes Mellitus: A Pragmatic Guide for Diagnosis, Management, and Care [47]	The International Federation of Gynecology and Obstetrics (FIGO)	2015	International	
21	Screening, Diagnosis and Management of Diabetes in Pregnant Women: National Guideline, Sri Lanka [48]	Ministry of Health, Government of Sri Lanka	2014	Sri Lanka	Lower-middle-income
22	Clinical Practice Guidelines: Diabetes Mellitus [49]	Ministry of Health, Singapore	2014	Singapore	High Income
23	Screening, Diagnosis and Management of Gestational Diabetes in New Zealand: A clinical practice guideline [50]	New Zealand Ministry of Health	2014	New Zealand	High Income
24	Clinical Practice Guidelines for Diabetes Management in Nigeria [51]	Diabetes Association of Nigeria	2013	Nigeria	Lower-middle-income
25	Indonesian Clinical Practice Guidelines for Diabetes in Pregnancy [52]	Indonesian Society of Endocrinology/ Indonesian Task Force on Reproductive Diseases	2013	Indonesia	Lower-middle-income
26	Diabetes and Pregnancy: An Endocrine Society Clinical Practice Guideline [53]	Endocrine Society	2013	USA	High Income

ACOG - American College of Obstetricians and Gynecologists; ADA – American Diabetes Association; FIGO – International Federation of Gynecology and Obstetrics; HKCOG - Hong Kong College of Obstetricians and Gynaecologists; SAFES - South Asian Federation of Endocrine Societies; SEMSDA - Society for Endocrinology, Metabolism and Diabetes of South Africa; SEMT - Society of Endocrinology and Metabolism of Turkey; SIGN – Scottish Intercollegiate Guidelines Network

### Quality of guidelines

Eight [28–30, 32, 41, 42, 44, 50] of the 26 appraised CPGs (31%) were assessed as high quality, 6 (23%)[31, 37, 38, 43, 49, 53] as moderate and 12 (46%)[33– 36, 39, 40, 45–48, 51, 52] as low quality (Table 2). The highest scoring domains were scope and purpose (98%) and clarity of presentation (98%). Editorial independence was the lowest scoring domain (47%) followed by rigor of development (54%). The Scottish Intercollegiate Guidelines Network (SIGN) CPG [42] was the most comprehensive and the Ministry of Health, Sri Lanka CPG [48] was the least comprehensive.

CPGs from low-and-middle-income countries (LMICs) scored lower across all the domains of the AGREE II tool than those from high income countries (HICs). Only one CPG from LMICs was assessed as high quality (Malaysia) and two were assessed as moderate quality (South Africa and The Philippines), with the remaining 7 as low quality. Similar to CPGs from HICs, the highest scoring domain was clarity of presentation but CPGs from LMICs scored lowest in the “rigor of development” domain. The other prioritized domain of “applicability” was also lower in CPGs from LMICs compared to HICs.

**Table 2:** AGREE II assessment of included guidelines

Guideline title	Domain 1. Scope and Purpose	Domain 2. Stakeholder Involvement	Domain 3. Rigor of Development	Domain 4. Clarity of Presentation	Domain 5. Applicability	Domain 6. Editorial Independence	Overall Assessment	Guideline quality
ADA 2023	100%	100%	97%	100%	90%	92%	7	High
NICE 2015	100%	100%	100%	100%	100%	75%	7	High
New Zealand 2014	100%	89%	86%	100%	100%	50%	7	High
Canada 2019	100%	94%	95%	100%	96%	92%	7	High
Malaysia 2017	100%	61%	99%	100%	88%	75%	7	High
Scotland 2017	100%	100%	100%	100%	100%	100%	7	High
Qatar 2021	100%	83%	83%	100%	73%	75%	6	High
Germany 2019	97%	67%	91%	100%	38%	100%	6	High
South Africa 2017	97%	78%	69%	100%	77%	83%	5	Moderate
RACGP 2020	100%	44%	64%	100%	100%	50%	5	Moderate
Endocrine 2013	100%	64%	66%	97%	10%	46%	4	Moderate
Philippines 2018	92%	56%	57%	100%	19%	25%	4	Moderate
Singapore 2014	97%	92%	53%	100%	94%	0%	4	Moderate
SAFES 2018	94%	53%	5%	100%	56%	0%	3	Low
FIGO 2015	97%	53%	46%	97%	40%	63%	4	Low
India 2018	97%	44%	5%	97%	83%	0%	4	Low
Nigeria 2013	100%	64%	30%	97%	98%	17%	4	Low
Iran 2021	97%	64%	35%	100%	17%	92%	3	Low
Turkey 2019	100%	50%	48%	100%	85%	42%	3	Low
Indonesia 2013	97%	61%	19%	86%	19%	83%	3	Low
Oman 2015	100%	56%	14%	97%	42%	0%	3	Low
ACOG 2017	97%	36%	65%	100%	35%	17%	3	Low
Sri Lanka 2014	89%	14%	5%	94%	8%	0%	2	Low
Poland 2018	97%	50%	30%	94%	25%	0%	2	Low
Greece 2020	97%	50%	24%	97%	35%	33%	2	Low
HKCOG 2016	94%	42%	11%	92%	13%	0%	2	Low
Average (Total)	98%	64%	54%	98%	59%	47%		
Average (LMICs)	96%	55%	37%	97%	55%	42%		Low
Average (HICs)	99%	71%	65%	98%	63%	49%		Moderate

ACOG - American College of Obstetricians and Gynecologists; ADA – American Diabetes Association; FIGO – International Federation of Gynecology and Obstetrics; HIC – high income countries; HKCOG - Hong Kong College of Obstetricians and Gynaecologists; LMIC – low-and-middle-income countries; RACGP – Royal Australian College of General Practitioners; SAFES - South Asian Federation of Endocrine Societies; SEMSDA - Society for Endocrinology, Metabolism and Diabetes of South Africa; SEMT - Society of Endocrinology and Metabolism of Turkey; SIGN – Scottish Intercollegiate Guidelines Network

### Evidence base for postnatal care recommendations

All 26 CPGs [28–53] had recommendations for postpartum glucose screening, 25 (96%)[28–38,40–53] recommended lifestyle management advice, 23 (88%)[28–31, 33, 35–39, 41–53] recommended breastfeeding, 16 (62%)[28, 30, 33, 36–38, 42–44, 46–50, 52, 53] recommended contraception and family planning advice, 24 (92%)[28–40, 42–46, 48–50, 52, 53] recommended long term glucose screening, and only 3 (12%) [28, 29, 38] gave recommendations about mental health. Nine CPGs did not report using an evidence appraisal tool [33, 36, 38, 39, 45, 46, 48, 51, 52], five used GRADE [37, 47, 49, 50, 53], and twelve [28–32, 34, 35, 40–44] used different grading frameworks including from the US Preventive Services Task Force [40, 44], American Diabetes Association [28], National Institute for Health and Care Excellence [32], National Health and Medical Research Council [31], American College of Physicians [34], Scottish Intercollegiate Guidelines Network [29, 42], and Strength of Recommendation Taxonomy [43]. Eleven CPGs [33, 36, 38, 39, 43, 45–48, 51, 52] did not provide any supporting evidence for their recommendations.

#### Postpartum glucose screening

All twenty-six CPGs recommended testing within the first 6 months after birth to detect impaired glucose tolerance (Table 3). The earliest recommendation was at 4 weeks (4–6 weeks by 6 CPGs) [28, 30, 34, 37, 40] and the latest was up to 6 months [41], with most recommending 6–12 weeks. Almost all CPGs recommended a 75 g 2 h oral glucose tolerance test (OGTT) as the best screening test, except for the NICE and SIGN CPGs which recommended fasting blood glucose (FBG) [32, 42], and the New Zealand CPG which recommended Hb1Ac [50]. Most of the recommendations were strong (*n* = 15) but had a low or very low certainty of evidence (*n* = 16). Recommendations of six CPGs [30, 31, 35, 41, 42, 50] were based on expert consensus. Only three CPGs [41, 42, 50] recommended reminders to improve uptake of postpartum glucose screening, all weak recommendations with low certainty of supporting evidence.

#### Breastfeeding

Twenty-three CPGs [28–31, 33, 35–39, 41–53] recommended breastfeeding in the immediate postpartum period for women who had GDM (Table 4). Only eight of these [29, 30, 35, 39, 41, 44, 50, 51] specified the duration of breastfeeding (range 3-6months). Fourteen CPGs gave a strong recommendation for breastfeeding [28–30, 33, 35–39, 43, 45, 47, 48, 53] while nine [31, 41, 42, 44, 46, 49–52] gave a weak recommendation. Almost half of the CPGs with breastfeeding recommendations did not report the supporting evidence base. Of those [11, 28, 29, 35, 37, 41,42, 44, 47, 49, 50, 53] reporting an evidence base, five reported evidence of moderate certainty [28, 29, 44, 47, 53], one reported evidence of high certainty [37], and five had a low/ very low certainty of supporting evidence [35, 41, 42, 49, 50] with three being expert consensus [35, 42, 50].

#### Lifestyle management

All but one CPG [39] recommended lifestyle advice with the aim of maintaining a healthy body weight in the postnatal period (Table 4). Twenty CPGs [28–38, 40, 41, 43–48, 53] gave a strong recommendation, with nine CPGs [28, 29, 31, 32, 34, 43, 44, 47, 50] basing their recommendations on high certainty evidence including randomized controlled trials (RCTs). Six CPGs [30, 35, 37, 40, 49, 53] based their recommendations on very low certainty evidence, including expert opinion, and eight [33, 36, 38, 45, 46, 48, 51, 52] did not report any supporting evidence. Eight CPGs [28, 30, 35, 38, 40, 43, 44, 50] recommended metformin in addition to lifestyle advice to delay or prevent the onset of diabetes in the presence of persistent impaired glucose tolerance in the postnatal period. All but one CPG [50] provided this as a strong recommendation and most recommendations were based on high certainty evidence.

#### Family planning/ contraception

Sixteen CPGs [28, 30, 33, 36–38, 42–44, 46–50, 52, 53] provided recommendations on family planning (Table 5). Six [33, 36, 38, 43, 46, 48] reported no supporting evidence, two [47, 52] reported very low certainty of supporting evidence, three reported evidence of low certainty [30, 44, 49], and two CPGs recommendations were based on expert consensus [42, 50]. The ADA [28] was the only CPG to report supporting evidence of high certainty which included a systematic review, and the CPGs from the Philippines [37] and the Endocrine Society [53] reported evidence of moderate certainty. Seven CPGs [28, 30, 33, 36, 43, 47, 53] provided strong recommendations with the remaining nine [37, 38, 42, 44, 46, 48–50, 52] providing weak recommendations.

#### Long-term follow up care

All the CPGs recommended long term follow up of women who experience GDM due to the increased future risk of developing impaired glucose tolerance (Table 5). Twenty-three CPGs [28–40, 42–46, 48–50, 52, 53] specified the frequency of ongoing glucose surveillance (every 1–3 years) using different glucose screening tests. Of the sixteen CPGs that specified the type of screening test [28–34, 36–39, 42, 43, 48, 50, 52], HbA1c was most commonly recommended [28–32, 38, 42, 43, 50]. Nineteen CPGs [28–40, 43–46, 48, 53] provided a strong recommendation, with ten CPGs [30, 33, 36, 38, 39, 44–46, 48, 52] not reporting the certainty of evidence and nine reporting low or very low certainty of evidence [31, 32, 34, 35, 40, 42, 49, 50, 53] used in making the recommendations. Only three CPGs [38, 43, 50] recommended cardiovascular/ metabolic syndrome screening in the postnatal period. Two of these were strong recommendations [38, 43] and only one had a high certainty evidence base [43].

#### Psychological care

Only three CPGs [28, 29, 38] provided recommendations for maternal postnatal mental health (Table 5). The ADA provided a strong recommendation based on expert opinion to include psychosocial assessment in GDM postnatal care [28], and the German Diabetes Association provided a strong recommendation based on moderate certainty of evidence to assess maternal mental well-being at 6–12 weeks using the EPDS tool [29]. The third recommendation provided by the South Asian Federation of Endocrine Societies was conditional for women who experienced fetal loss to have their psychological well-being assessed (strong recommendation with no supporting evidence) [38].

**Table 3:** Comparison of postpartum glucose screening recommendations across different CPGs

Guideline	PPG screening recommended	Timing (weeks)	Recommended screening test	Threshold for PPG test interpretation specified	Threshold for IGT/ pre-diabetes	Threshold for T2DM	Reminders recommended for PPG screening
**New Zealand 2014**	Yes	12	HbA1c	Yes	41–49 mmol/mol	≥ 50 mmol/mol	Yes
**Sri Lanka 2014**	Yes	6–8	OGTT	Yes	5.5 mmol/L (100 mg/dL)		No
**ADA 2023**	Yes	4–12	OGTT	Yes	7.8–11 mmol/L (140-199 mg/dL)	≥ 11.1 mmol/L (200 mg/dL)	No
**Malaysia 2017**	Yes	6	OGTT	No	-	-	No
**India 2018**	Yes	6	OGTT	Yes	140-199 mg/dL	≥ 200 mg/dL	No
**NICE 2015**	Yes	6–13	FPG or HbA1c	Yes	6-6.9 mmol/L	≥ 7.0 mmol/L	No
**Qatar 2021**	Yes	4–12	OGTT	No	-	-	No
**FIGO 2015**	Yes	6–12	OGTT	Yes	7.8-11.1mmol/L	≥ 11.1 mmol/L	No
**Endocrine Society 2013**	Yes	6–12	OGTT	No	-	-	No
**Canada 2018**	Yes	6–36	OGTT	No	-	-	Yes
**Iran 2021**	Yes	4–12	OGTT	No	-	-	No
**Philippines 2018**	Yes	4–12	OGTT	Yes	7.8–11.0 mmol/L (140-199 mg/dL)	≥ 11.1 mmol/L (200 mg/dL)	No
**Oman 2015**	Yes	6–12	OGTT	Yes	5.5–6.9 mmol/L (100-125 mg/dL)	≥ 11.1 mmol/L (200 mg/dL)	No
**SEMDSA 2017**	Yes	6	OGTT	No	-	-	No
**Indonesia 2013**	Yes	6–12	OGTT	No	-	-	No
**Germany 2018**	Yes	6–12	OGTT	Yes	7.8–11.0 mmol/L (140-199 mg/dL)	≥ 11.1 mmol/L (200 mg/dL)	No
**Singapore 2014**	Yes	6–12	OGTT	No	-	-	No
**Greece 2020**	Yes	8–12	OGTT	No	-	-	No
**SIGN 2017**	Yes	6	FPG and OGTT	No	-	-	Yes
**Australia (RACGP) 2020**	Yes	6–12	OGTT	No	-	-	No
**SEMT 2019**	Yes	4–12	OGTT	No	-	-	No
**ACOG 2017**	Yes	4–12	FPG or OGTT	Yes	140–199 (OGTT) 100–125 (FPG)	> 199 (OGGT) > 125 (FPG)	No
**SAFES 2018**	Yes	6	OGTT	Yes		≥ 11.1 mmol/L (WHO criteria)	No
**Polish 2018**	Yes	6	OGTT	Yes	7.8-11.1mmol/L (WHO criteria)	≥ 11.1 mmol/L (WHO criteria)	No
**Nigeria 2013**	Yes	6–12	OGTT	Yes	7.8-11.1mmol/L (WHO criteria)	≥ 11.1 mmol/L (WHO criteria)	No
**HKCOG 2016**	Yes	6–12	OGTT or HbA1c	No	-	-	No

ACOG - American College of Obstetricians and Gynecologists; ADA – American Diabetes Association; FIGO – International Federation of Gynecology and Obstetrics; FPG – fasting plasma glucose; HbA1c – haemoglobin A1c; HKCOG - Hong Kong College of Obstetricians and Gynaecologists; IGT – impaired glucose tolerance; NICE – National Institute for Health and Care Excellence; OGTT – oral glucose tolerance test; PPG – postpartum glucose; RACGP – Royal Australian College of General Practitioners; SAFES - South Asian Federation of Endocrine Societies; SEMSDA - Society for Endocrinology, Metabolism and Diabetes of South Africa; SEMT - Society of Endocrinology and Metabolism of Turkey; SIGN – Scottish Intercollegiate Guidelines Network; T2DM – type 2 diabetes mellitus; WHO – World Health Organization

**Table 4:** Comparison of lifestyle advice, breastfeeding, and contraception recommendations across different CPGs

Guideline	Lifestyle advice recommended	Type of lifestyle advice recommended	Counselling on future risk of impaired glucose tolerance	Contraception recommended	Breastfeeding recommended	Duration of breastfeeding specified	Recommendation for blood glucose monitoring immediately after delivery	Recommendation for discontinuing medications
**New Zealand 2014**	Yes	Diet; Exercise	Yes	Yes	Yes	Yes	Yes	Yes
**Sri Lanka 2014**	Yes	Diet; Exercise; Weight Control	No	Yes	Yes	No	Yes	Yes
**ADA 2023**	Yes	Diet; Exercise; Weight Control	No	Yes	Yes	No	No	Yes
**Malaysia 2017**	Yes	Diet; Exercise; Weight Control	No	Yes	Yes	Yes	No	Yes
**India 2018**	Yes	Exercise; Weight Control	No	Yes	Yes	No	No	No
**NICE 2015**	Yes	Diet; Exercise; Weight Control	Yes	No	No	No	No	Yes
**Qatar 2021**	Yes	Diet; Exercise; Weight Control	Yes	Yes	Yes	Yes	No	Yes
**FIGO 2015**	Yes	Diet; Exercise; Weight Control	No	Yes	Yes	No	No	No
**Endocrine Society 2013**	Yes	Diet; Exercise	Yes	Yes	Yes	No	Yes	Yes
**Canada 2018**	Yes	Diet; Exercise; Weight Control	Yes	No	Yes	Yes	No	No
**Iran 2020**	Yes	Diet; Exercise	No	No	No	No	No	No
**Philippines 2018**	Yes	Diet; Exercise; Weight Control	No	Yes	Yes	No	Yes	Yes
**Oman 2015**	Yes	Exercise; Weight Control	Yes	Yes	Yes	No	Yes	No
**SEMDSA 2017**	Yes	Diet; Exercise; Weight Control	No	Yes	Yes	No	No	Yes
**Indonesia 2013**	Yes	Diet	No	Yes	Yes	No	Yes	No
**Germany 2018**	Yes	Diet; Exercise; Weight Control	No	No	Yes	Yes	No	No
**Singapore 2014**	Yes	Diet; Exercise; Weight Control	No	Yes	Yes	No	Yes	Yes
**Greece 2020**	Yes	Diet; Exercise; Weight Control	No	Yes	Yes	No	Yes	Yes
**SIGN 2017**	Yes	Diet; Exercise; Weight Control	No	Yes	Yes	No	No	No
**Australia (RACGP) 2020**	Yes	Exercise; Weight Control	Yes	No	Yes	No	No	No
**SEMT 2019**	Yes	Diet; Exercise; Weight Control	No	No	Yes	Yes	No	No
**ACOG 2017**	Yes	Diet; Exercise; Weight Control	No	No	No	No	No	No
**SAFES 2018**	Yes	Diet; Weight Control	No	Yes	Yes	No	Yes	Yes
**Poland 2018**	No	-	No	No	Yes	Yes	Yes	Yes
**Nigeria 2013**	Yes	Diet; Exercise	No	No	Yes	Yes	No	No
**Hong Kong 2016**	Yes	Diet; Exercise; Weight Control	Yes	No	Yes	No	No	No

ACOG - American College of Obstetricians and Gynecologists; ADA – American Diabetes Association; FIGO – International Federation of Gynecology and Obstetrics; HKCOG - Hong Kong College of Obstetricians and Gynaecologists; NICE – National Institute for Health and Care Excellence; RACGP – Royal Australian College of General Practitioners; SAFES - South Asian Federation of Endocrine Societies; SEMSDA - Society for Endocrinology, Metabolism and Diabetes of South Africa; SEMT - Society of Endocrinology and Metabolism of Turkey; SIGN – Scottish Intercollegiate Guidelines Network

**Table 5:** Comparison of long-term follow-up care recommendations across different CPGs

Guideline	Long term follow-up recommended	Long term glucose testing recommended	Frequency of testing	Recommended screening test	Screening for cardiovascular and/or metabolic disorders recommended	Psychosocial support recommended
**New Zealand 2014**	Yes	Yes	Annually	HbA1c	Conditional	No
**Sri Lanka 2014**	Yes	Yes	Annually	FPG	No	No
**ADA 2023**	Yes	Yes	1–3 years	Any (HbA1c, FPG or OGTT)	No	Yes
**Malaysia 2017**	Yes	Yes	Annually	Not specified	No	No
**India 2018**	Yes	Yes	Annually	OGTT	No	No
**NICE 2015**	Yes	Yes	Annually	HbA1c	No	No
**Qatar 2021**	Yes	Yes	Annually	HBA1c or FPG	No	No
**FIGO 2015**	Yes	No	-	-	No	No
**Endocrine Society 2013**	Yes	Yes	Not specified (periodically as well as before future pregnancies).	Not specified	No	No
**Canada 2018**	Yes	No	-	-	No	No
**Iran 2020**	Yes	Yes	Annually	FPG	No	No
**Philippines 2018**	Yes	Yes	1–3 years	Any (HBA1c, FPG or OGTT)	No	No
**Oman 2015**	Yes	Yes	Normal result: 2 yearly IGT: Annually	Not specified	No	No
**SEMDSA 2017**	Yes	Yes	Annually	HbA1c	No	No
**Indonesia 2013**	Yes	Yes	1–3 years	OGTT	No	No
**Germany 2018**	Yes	Yes	Annually/2 years	Annually (FPG or HbA1c), 2 yearly (OGTT)	No	Yes
**Singapore 2014**	Yes	Yes	3 years	Not specified	No	No
**Greece 2020**	Yes	Yes	1–3 years	OGTT	No	No
**SIGN 2017**	Yes	Yes	Annually	FPG or HbA1c	No	No
**Australia (RACGP) 2020**	Yes	Yes	3 years	HBA1c or FPG	No	No
**SEMT 2019**	Yes	Yes	When planning a pregnancy	Not specified	No	No
**ACOG 2017**	Yes	Yes	1–3 years	Not specified	No	No
**SAFES 2018**	Yes	Yes	Annually	OGTT or HbA1c	Yes	Yes
**Poland 2018**	Yes	Yes	Annually	OGTT	No	No
**Nigeria 2013**	Yes	Yes	Not specified	Not specified	No	No
**HKCOG 2016**	Yes	Yes	Higher risk (annually) Lower risk (every 3years)	Not specified	No	No

ACOG - American College of Obstetricians and Gynecologists; ADA – American Diabetes Association; FIGO – International Federation of Gynecology and Obstetrics; FPG – fasting plasma glucose; HbA1c – haemoglobin A1c; HKCOG - Hong Kong College of Obstetricians and Gynaecologists; IGT - impaired glucose tolerance; NICE – National Institute for Health and Care Excellence; OGTT – oral glucose tolerance test; RACGP – Royal Australian College of General Practitioners; SAFES - South Asian Federation of Endocrine Societies; SEMSDA - Society for Endocrinology, Metabolism and Diabetes of South Africa; SEMT - Society of Endocrinology and Metabolism of Turkey; SIGN – Scottish Intercollegiate Guidelines Network

### Research gaps in recommendations

The research gaps identified by CPGs, and those identified by low/very low certainty or no evidence were similar across the different domains, and included optimum timing and diagnostic test for postpartum glucose screening, optimum duration of breastfeeding, and evaluation of effective uptake of lifestyle interventions (Table 6). The research gaps identified only by low/very low certainty of evidence or expert consensus included contraception recommendations, optimum frequency for long-term glucose screening, and the need for postpartum metabolic screening.

**Table 6:** Research gaps in the postnatal care recommendations of GDM care

Topic	Research gaps identified by CPGs	Research gaps identified by low/ very low certainty of evidence	Research gaps identified by expert opinion/ consensus
**Postpartum glucose screening**	Why do women not engage with postnatal glucose tolerance testing? [32]	What is the most accurate diagnostic test for hyperglycaemia postpartum? What should be the timing of postnatal glucose testing? [29, 30, 32, 34, 38, 39,44, 47, 49, 52]	What is the most accurate test for detecting hyperglycaemia postpartum? What is the optimal timing of postpartum glucose testing? [31, 33, 35, 36, 40– 42, 45, 46, 48, 50, 51]
	What is the efficacy of HbA1c test for detecting diabetes and/or glucose intolerance in the postnatal period? [32]	What should women with gestational diabetes and their providers be reminded about the timing of postpartum screening? [50]	At what threshold value should women be referred to a specialist? [48]
	What are the reasons for non-adherence to postpartum screening? [50] Can follow-up visits and care be linked to the child’s vaccination program and well-baby clinics? [47]	What are some methods that can be undertaken to improve postpartum testing to women with a history of GDM? [41]	Where should women with impaired fasting glucose, impaired glucose tolerance, or diabetes be referred to? [36, 40]
**Immediate postpartum**	What is the best test for detecting impaired glucose intolerance in the immediate postpartum period? [32]	How should the dramatic decrease in insulin resistance be managed for the initial few days postpartum? [28]	Should the blood glucose of women be routinely monitored in the immediate postpartum period? When and how long should this be for? What is the normal range of blood glucose levels? [33, 39, 46, 50]
	Are there effective long-term pharmacological interventions that can be recommended postnatally for women who have been diagnosed with gestational diabetes to prevent the onset of type 2 diabetes? [32]	Should blood glucose-lowering medication be continued for women with GDM following delivery? [38, 44, 53]	Should diabetes medication for women with a GDM diagnosis be continued or discontinued after birth? [33, 39,49, 50]
		What is the optimal timing for postpartum glucose screening in the immediate postpartum period? [38, 52, 53]	If women with GDM are given insulin in the antenatal period, how should their dose be adjusted postpartum? [48]
**Breastfeeding**	What is the optimum duration of breastfeeding? [44]	What is the optimum timing and duration of breastfeeding by mothers with GDM? [38, 47]	What is the optimum timing and duration of breastfeeding by mothers with GDM? [33, 35, 36, 39, 41–43, 45, 46, 51]
	Does breastfeeding influence the risk of subsequent obesity or DM specifically in the offspring of women with GDM? [37]	What breastfeeding advice is recommended for women with GDM? [49, 52] What advice on breastfeeding should be provided to obese women with GDM? [29]	What should women be encouraged about breastfeeding and skin to skin contact after birth? [50]
**Lifestyle management**	Does the diagnosis of impaired glucose tolerance influence the uptake of lifestyle changes after birth in a woman with previous GDM? [32]	What postpartum lifestyle behaviours should be recommended to women with prior GDM to reduce GDM recurrence rate in subsequent pregnancies as well as risk of type 2 diabetes? [37, 38,41, 47, 52, 53]	What lifestyle behaviours should women with GDM be counselled about postpartum and before the next pregnancy? [28, 33, 35, 36,45, 48, 49, 51]
	Randomised controlled trials that evaluate the outcomes of lifestyle versus pharmacological interventions to prevent type 2 diabetes in women with a previous history of GDM. [50]	What postpartum lifestyle modifications and pharmacological interventions should be recommended to women with prior GDM who have impaired glucose tolerance postpartum? [38, 40, 46]	What lifestyle advice should patients with hyperglycaemia receive regarding exercise and weight control? [46]
**Contraception**		What contraceptive methods are recommended for women with previous GDM? [38, 47, 49]	What contraceptive methods are recommended for women with previous GDM? [33, 42, 46, 48, 50]
**Long-term follow up care**	There should be research to address the knowledge gaps to better understand the links between maternal health and noncommunicable diseases (best practice standards for testing, management, and care of women with GDM), including cost-effectiveness models…to make the best choices for testing and management of GDM given country specific burden of disease and resources. [47]	What should be the timing for long-term glucose screening in women with prior GDM? What is the most accurate diagnostic test for this? [31, 38]	What should be the timing for long-term glucose screening in women with prior GDM? [33, 35, 36, 40, 42, 49, 50] How does the timing differ between low-risk vs. high-risk women? [45]
	There is no clear guidance about the type of tests (should these women undergo annual OGTTs or can fasting plasma glucose or HbA1c measurement suffice?) or the frequency and duration for ongoing surveillance. [42, 47]	If the result of (e.g. 6 week) postpartum glucose testing is negative, when should long-term glucose screening be performed? [52, 53]	For women with GDM who screen negative at the 6-week postnatal glucose test, what information about further screening and lifestyle interventions should be provided to them? [48]
		Should metabolic screening be offered after delivery to women with GDM? [38]	How often should glycaemic status of all women with GDM be assessed in the long-term following the 6–12 weeks test? [46]
**Psychological care**		How is a depressive mood for women with GDM clarified postpartum and what actions must be taken? [29]	How can mental health issues for women with GDM be prevented or managed postpartum? [28]
		How should the psychological wellbeing of mothers with GDM who experience fetal loss be managed? [38]	

CPG – clinical practice guideline; DM - diabetes mellitus; GDM – gestational diabetes mellitus; HbA1c – haemoglobin A1c; OGTT – oral glucose tolerance test

## Discussion

GDM has significant long-term implications for maternal health. We sought to examine postnatal care recommendations after GDM, the certainty of supporting evidence for the recommendations (including strength of recommendation), and evidence gaps which may need further research. Almost half of the twenty-six included CPGs were appraised as being of low-quality and a third were of high quality. Most of the reported evidence was of low/ very low certainty and several CPGs based their recommendations on expert opinion. We also identified several evidence gaps across all the postnatal care domains we assessed, with the most gaps pertaining to postnatal glucose screening.

A previous study assessing dietary recommendations for women with GDM identified only 10% of guidelines as high quality, with two-thirds being of low quality [27]. In that study, all domains of the AGREE II tool were given equal importance and a score of ≥ 70% was required across all the domains for a guideline to be deemed high quality. In our study we prioritized domains 3 (rigor of development) and 5 (applicability) to deem a CPG high quality which could explain the difference in results. An interplay of different aspects of a CPG has been reported to determine the uptake of recommendations by intended users. The strength of the evidence base (relating to the “rigor of development” domain) and ease of dissemination and implementation (as reflected the “applicability” domain) have been highlighted as important to recommendation uptake [54]. In our review the average score for the applicability domain of 59% is of concern as several studies have highlighted the importance of context specific dissemination resources and practice guides for the uptake of guideline recommendations by health professionals [54, 55]. 

Most of the postnatal care recommendations were similar in different CPGs across the domains we assessed (postpartum glucose screening, lifestyle management, breastfeeding, family planning and long term follow up care). However, important variations were found in the recommendations regarding long-term glucose screening tests, and frequency of follow up care.

Although postpartum glucose screening within 6 months after birth was recommended by all the CPGs, only three recommended ways to improve uptake of the screening. Generally, uptake of postpartum glucose screening has been suboptimal, with only half of women attending the screening [56]. Significantly, women who have higher future risk of type 2 diabetes and perinatal depression have been found to engage least with screening [15]. Interventions that use proactive patient contact approaches such as phone calls or sending reminders have been reported to increase uptake of postpartum glucose screening [57, 58] and could be recommended in this context. Some studies have also identified non-standardised coordination of care during the transition from antenatal specialist care to postnatal primary care as a significant barrier to uptake of postpartum glucose screening and long-term GDM follow up care [59, 60]. Three guidelines [32, 36, 38] recommended integration of postpartum care with other primary care services such as child health services and diabetes prevention programmes to help bridge this gap. Qualitative evidence suggests this integrative approach will be acceptable to mothers and healthcare providers [59].

Most CPGs recommended a 2 h OGTT as the test of choice for postpartum glucose screening, as this has been shown to better detect glucose impairment in the first year after the birth compared to other tests like the HbA1c [61]. However, qualitative studies have reported that the inconvenience associated with the OGTT contributes to low uptake of this test [62]. Two cohort studies have reported that fasting plasma glucose in addition to a lower cut-off for HbA1c may be an acceptable alternative to detecting glucose impairment during this period [63, 64]. However, higher quality studies are needed to determine the efficacy of these alternative tests.

In the long term, women with history of GDM have a 70% lifetime risk of developing type 2 diabetes [65]. Most CPGs, therefore, recommended glucose screening every 1–3 years for women with a history of GDM. However, there was little consensus in the recommended screening test, and these recommendations had very low certainty of supporting evidence. The FIGO guideline [47] also noted that postnatal care guidance is often glucose centric, missing out other important parameters. Consistent with this, we found few recommendations on screening for cardiovascular disease and other metabolic disorders in the postpartum period. A meta-analysis of data from more than 5 million women reported that women with GDM had a twofold higher risk of future cardiovascular events compared to women with no GDM, independent of the incident risk of type 2 diabetes [66]. This is an important gap, as recent evidence from the American Heart Association suggests that interventions implemented in response to postnatal metabolic screening can significantly improve cardiovascular health for women who experience adverse pregnancy outcomes [67]. These metabolic markers, including serum triglycerides and blood pressure measurements, have been reported to be elevated among women who experience GDM compared to women who have uncomplicated pregnancies [68], highlighting the importance of integration of metabolic screening in GDM postnatal care and CPGs.

Similarly, only three CPGs recommended screening for maternal mental health status in the postnatal period, despite evidence of an increased risk of postnatal depression among women with GDM [69].

Breastfeeding was strongly recommended by most guidelines, but there was uncertainty about the recommended duration of breastfeeding. Women with GDM have been reported to have lower rates and shorter duration of exclusive breastfeeding [70–72] despite evidence reporting significant benefits of increased duration of breastfeeding on neonatal metabolic outcomes and a reduction in maternal future risk of type 2 diabetes [73, 74]. A recent comprehensive review on facilitators of breastfeeding in women with GDM noted that social and family support were key to initiation and continuation of breastfeeding [75]. However, most recommendations in our systematic review did not specify how mothers can be supported to breastfeed outside of the immediate postpartum period.

Lifestyle advice (diet, exercise, and weight control) was strongly recommended in most CPGs, most based on high to moderate certainty of evidence. Although the effectiveness of lifestyle modification in improving glucose tolerance after GDM is clear, a systematic review reported low adherence to continued lifestyle modifications in women with GDM [76]. This low engagement has been attributed in part to lack of education by health professionals resulting in a low perceived risk of future complications [76, 77]. However, in our review, only a third of the CPGs assessed gave specific recommendations for healthcare professionals to provide postnatal counselling on the future risk of glucose impairment.

Recommendations for family planning and contraception were similar across the sixteen CPGs that recommended them. These emphasised shared decision making with women to ensure future pregnancies were appropriately planned. However, 80% of these CPGs provided recommendations which had no or low certainty of supporting evidence, and ten did not provide any recommendations on contraception. Of those that reported the evidence base, the studies cited were mostly carried out in women with Type 1 or type 2 diabetes and not exclusively in women with GDM. The only randomized trial reported in the supporting evidence was over three decades old and performed primarily in women of Latin American descent to assess the metabolic effect of oral contraceptives in women with previous GDM [78]. Considering the recent changes in diagnostic thresholds, increasing prevalence of GDM across different population groups globally, and increasing choice in contraceptives, newer high-quality evidence on contraceptives after GDM may be required. Additionally, some of the medical contraindications to hormonal contraceptives such as older age, cardiovascular risk factors, and high BMI [79], are also risk factors commonly found in women who develop GDM [4]. However, most CPGs’ recommendations did not reflect considerations of other medical risk factors or contraindications in their recommendations.

### Comparison between guidelines from different economy regions

More CPGs from LMICs were assessed as low quality compared to those from HICs. This is likely because most of the LMIC CPG recommendations were based on expert opinion, reported very little information on the guideline development process, and did not provide information on resource implications for implementing the recommendations. Lack of guidelines for use by first line health professionals has been reported as a challenge in GDM management in LMICs [80]. Our findings are in line with this report.

Generally, most of the postnatal recommendations were similar across CPGs from LMICs and HICs. However, none of the LMIC CPGs assessed provided recommendations on postnatal risk counselling, despite evidence reporting non-adherence to follow-up care by women in LMICs due to low level of knowledge and perceived risk of long-term complications associated with GDM [80]. 

Additionally, a higher number of CPGs from LMICs than from HICs reported no supporting evidence for recommendations. This lack of evidence affects the credibility of the guidelines as reflecting the best available evidence and ensuring recommendations accurately reflect benefits and harms [24]. Most CPGs from LMICs also cited their source of evidence as other CPGs from HICs. This is likely due to the large resources needed for de novo guideline development. To navigate this, there has been a body of research on frameworks to adapt guidelines to specific contexts [81]. However, none of the CPGs from LMICs which reported supporting evidence as other HIC CPGs reported an adaptation methodology in the guideline development process. Healthcare contexts in LMICs also differ significantly from HICs, so not all the recommendations made for HIC healthcare settings may be appropriate or feasible in resource constrained settings. This also resulted in CPGs from LMICs not being able to report research gaps as there was little systematic search of evidence in the guideline development process. Hence, most of the research gaps we identified were from HICs.

It is also worth noting that, irrespective of economy regions, health system characteristics including payment mechanisms (health insurance and degree of out-of-pocket payments) [82], and the level of coordination of care from the antenatal to postnatal period [59] are factors which significantly affect uptake of GDM postnatal care recommendations and are not easily compared across countries.

### Strengths and limitations

This systematic review provides in-depth analysis of recommendations for postnatal care after GDM. We assessed the certainty of the evidence base behind the recommendations, and identified research gaps that could be the focus of further research. Strengths of this review include systematic literature searching using broad search terms, inclusion of up-to-date recent CPGs with full versions, and consistent assessment using the GRADE and AGREE II frameworks.

However, we limited our review to CPGs which were published in English since 2013, which may not reflect all the relevant GDM guidelines. This was due to language barriers and the aim of capturing the most recent guidelines.

## Conclusions

Clinical practice guidelines play an important role in the provision of evidence-based care. We assessed 26 GDM CPGs from 22 countries and found almost half of them to be of low quality. Most of the postnatal recommendations assessed had a low or very low certainty of supporting evidence base, highlighting the minimal high certainty evidence around postnatal and long-term care after GDM. We also identified evidence gaps in recommendations which may benefit from further research with the aim of improving the certainty of evidence base in postnatal care after GDM. The quality and evidence base of recommendations from CPGs from LMICs was lower than those from HICs, indicating further efforts are needed to build capacity for guideline development and reporting in LMICs.

## Electronic supplementary material

Below is the link to the electronic supplementary material.

Supplementary Material 1

## Data Availability

The data and materials used in this study are available from the corresponding author on reasonable request. All data generated or analysed during this study are included in this published article [and its supplementary information files].

## Abbreviations

ACOG: American College of Obstetricians and Gynecologists
ADA: American Diabetes Association
AGREE: Appraisal of Guidelines, Research, and Evaluation
BMI: Body Mass Index
CPG: Clinical Practice Guideline
EPDS: Edinburgh Postnatal Depression Scale
FBG: Fasting Blood Glucose
FIGO: International Federation of Gynecology and Obstetrics
FPG: Fasting Plasma Glucose
GDM: Gestational Diabetes Mellitus
GRADE: Grading of Recommendations Assessment, Development and Evaluation
HIC: High-income countries
HKCOG: Hong Kong College of Obstetricians and Gynaecologists
IGT: Impaired glucose tolerance
LMIC: Low-and-middle-income countries
NICE: National Institute for Health and Care Excellence
OGTT: Oral Glucose Tolerance Test
PPG: Postpartum Glucose
PRISMA: Preferred Reporting Items for Systematic Reviews and Meta-Analyses
RACGP: Royal Australian College of General Practitioners
RR: Relative Risk
SAFES: South Asian Federation of Endocrine Societies
SEMSDA: Society for Endocrinology, Metabolism and Diabetes of South Africa
SEMT: Society of Endocrinology and Metabolism of Turkey
SIGN: Scottish Intercollegiate Guidelines Network
WHO: World Health Organization
