# Supplementary material for: Postnatal care after gestational diabetes – a systematic review of clinical practice guidelines
Source: BMC Pregnancy Childbirth. 2024 Nov 4;24:720. doi: 10.1186/s12884-024-06899-w (PMC11536828; doi:10.1186/s12884-024-06899-w)
Supplement: Supplementary file 1 — Supplementary Material 1 [file 12884_2024_6899_MOESM1_ESM.docx]

**SUPPORTING INFORMATION**

Table S1: PRISMA checklist

| **Section and Topic** | **Item #** | **Checklist item** | **Location where item is reported** |
| --- | --- | --- | --- |
| **TITLE** | | |  |
| Title | 1 | Identify the report as a systematic review. | Title page |
| **ABSTRACT** | | |  |
| Abstract | 2 | See the PRISMA 2020 for Abstracts checklist.  **Background:** Provide an explicit statement of the main objective the review addresses  **Methods**: Report eligibility criteria; information sources; methods of assessing risk of bias; methods of synthesising results  **Results:** Provide the total number of included studies and participants and summarise relevant characteristics of studies; present results for main outcomes  **Discussion**: Provide a brief summary of the limitation of the evidence included in the review; provide a general interpretation of the results and important implications | Abstract pp1-2 |
| **INTRODUCTION** | | |  |
| Rationale | 3 | Describe the rationale for the review in the context of existing knowledge. | Background pp3-4 |
| Objectives | 4 | Provide an explicit statement of the objective(s) or question(s) the review addresses. | pp4-5 |
| **METHODS** | | |  |
| Eligibility criteria | 5 | Specify the inclusion and exclusion criteria for the review and how studies were grouped for the syntheses. | Methods: eligibility criteria |
| Information sources | 6 | Specify all databases, registers, websites, organisations, reference lists and other sources searched or consulted to identify studies. Specify the date when each source was last searched or consulted. | Methods: search strategy and data sources |
| Search strategy | 7 | Present the full search strategies for all databases, registers and websites, including any filters and limits used. | Table S2 |
| Selection process | 8 | Specify the methods used to decide whether a study met the inclusion criteria of the review, including how many reviewers screened each record and each report retrieved, whether they worked independently, and if applicable, details of automation tools used in the process. | Methods: study selection and data extraction |
| Data collection process | 9 | Specify the methods used to collect data from reports, including how many reviewers collected data from each report, whether they worked independently, any processes for obtaining or confirming data from study investigators, and if applicable, details of automation tools used in the process. | Methods: study selection and data extraction |
| Data items | 10a | List and define all outcomes for which data were sought. Specify whether all results that were compatible with each outcome domain in each study were sought (e.g. for all measures, time points, analyses), and if not, the methods used to decide which results to collect. | Methods: data extraction and appraisal of CPGs |
|  | 10b | List and define all other variables for which data were sought (e.g. participant and intervention characteristics, funding sources). Describe any assumptions made about any missing or unclear information. | Methods: data extraction and appraisal of CPGs |
| Study risk of bias assessment | 11 | Specify the methods used to assess risk of bias in the included studies, including details of the tool(s) used, how many reviewers assessed each study and whether they worked independently, and if applicable, details of automation tools used in the process. | Methods: data extraction and appraisal of CPGs |
| Effect measures | 12 | Specify for each outcome the effect measure(s) (e.g. risk ratio, mean difference) used in the synthesis or presentation of results. | Methods: data extraction and appraisal of CPGs |
| Synthesis methods | 13a | Describe the processes used to decide which studies were eligible for each synthesis (e.g. tabulating the study intervention characteristics and comparing against the planned groups for each synthesis (item #5)). | Methods: data extraction and appraisal of CPGs |
|  | 13b | Describe any methods required to prepare the data for presentation or synthesis, such as handling of missing summary statistics, or data conversions. | Methods: data extraction and appraisal of CPGs. Table S3 and S4 |
|  | 13c | Describe any methods used to tabulate or visually display results of individual studies and syntheses. | Methods: data extraction and appraisal of CPGs. Table S3 and S4 |
|  | 13d | Describe any methods used to synthesize results and provide a rationale for the choice(s). If meta-analysis was performed, describe the model(s), method(s) to identify the presence and extent of statistical heterogeneity, and software package(s) used. | Methods: data extraction and appraisal of CPGs. Table S3 and S4 |
|  | 13e | Describe any methods used to explore possible causes of heterogeneity among study results (e.g. subgroup analysis, meta-regression). | Methods: data extraction and appraisal of CPGs. |
|  | 13f | Describe any sensitivity analyses conducted to assess robustness of the synthesized results. | Methods: data extraction and appraisal of CPGs. Table S3 and S4 |
| Reporting bias assessment | 14 | Describe any methods used to assess risk of bias due to missing results in a synthesis (arising from reporting biases). | Methods: data extraction and appraisal of CPGs. |
| Certainty assessment | 15 | Describe any methods used to assess certainty (or confidence) in the body of evidence for an outcome. | Methods: data extraction and appraisal of CPGs. Table S3 and S4 |
| **RESULTS** | | |  |
| Study selection | 16a | Describe the results of the search and selection process, from the number of records identified in the search to the number of studies included in the review, ideally using a flow diagram. | Results. Figure 1: PRISMA diagram |
|  | 16b | Cite studies that might appear to meet the inclusion criteria, but which were excluded, and explain why they were excluded. | Figure 1: PRISMA diagram |
| Study characteristics | 17 | Cite each included study and present its characteristics. | Table 1 |
| Risk of bias in studies | 18 | Present assessments of risk of bias for each included study. | Table 2 |
| Results of individual studies | 19 | For all outcomes, present, for each study: (a) summary statistics for each group (where appropriate) and (b) an effect estimate and its precision (e.g. confidence/credible interval), ideally using structured tables or plots. | Tables 3-6 |
| Results of syntheses | 20a | For each synthesis, briefly summarise the characteristics and risk of bias among contributing studies. | Results: quality of guidelines, Evidence base for postnatal care recommendations, and research gaps in recommendations |
|  | 20b | Present results of all statistical syntheses conducted. If meta-analysis was done, present for each the summary estimate and its precision (e.g. confidence/credible interval) and measures of statistical heterogeneity. If comparing groups, describe the direction of the effect. | Results: quality of guidelines, evidence base for postnatal care recommendations, and research gaps in recommendations |
|  | 20c | Present results of all investigations of possible causes of heterogeneity among study results. | Results: quality of guidelines, Evidence base for postnatal care recommendations, and research gaps in recommendations. Table S5 |
|  | 20d | Present results of all sensitivity analyses conducted to assess the robustness of the synthesized results. | Results: quality of guidelines, Evidence base for postnatal care recommendations, and research gaps in recommendations. Table S5 |
| Reporting biases | 21 | Present assessments of risk of bias due to missing results (arising from reporting biases) for each synthesis assessed. | Results: Evidence base for postnatal care recommendations, and research gaps in recommendations. Table 3-5; S5 |
| Certainty of evidence | 22 | Present assessments of certainty (or confidence) in the body of evidence for each outcome assessed. | Results: Evidence base for postnatal care recommendations, and research gaps in recommendations. Table 3-5; S5 |
| **DISCUSSION** | | |  |
| Discussion | 23a | Provide a general interpretation of the results in the context of other evidence. | Discussion |
|  | 23b | Discuss any limitations of the evidence included in the review. | Discussion |
|  | 23c | Discuss any limitations of the review processes used. | Discussion: Strengths and limitations |
|  | 23d | Discuss implications of the results for practice, policy, and future research. | Discussion and conclusion |
| **OTHER INFORMATION** | | |  |
| Registration and protocol | 24a | Provide registration information for the review, including register name and registration number, or state that the review was not registered. | Methods: Protocol and registration |
|  | 24b | Indicate where the review protocol can be accessed, or state that a protocol was not prepared. | Methods: Protocol and registration |
|  | 24c | Describe and explain any amendments to information provided at registration or in the protocol. | N/A |
| Support | 25 | Describe sources of financial or non-financial support for the review, and the role of the funders or sponsors in the review. | Funding: pp 32 |
| Competing interests | 26 | Declare any competing interests of review authors. | Competing interest: pp 32 |
| Availability of data, code and other materials | 27 | Report which of the following are publicly available and where they can be found: template data collection forms; data extracted from included studies; data used for all analyses; analytic code; any other materials used in the review. | Supplementary data |

Table S2: Search Strategy

| Search database | Search strategy |
| --- | --- |
| Ovid Medline | 1) Mesh term [/Diabetes, Gestational/]  2) ("gestational diabetes" or gdm or "gestational diabetes mellitus" or "diabetes in pregnancy" or "diabetes in pregnancy" or “hyperglycaemia” or “glucose intolerance” or “insulin resistance”).mp. [mp=title, book title, abstract, original title, name of substance word, subject heading word, floating sub-heading word, keyword heading word, organism supplementary concept word, protocol supplementary concept word, rare disease supplementary concept word, unique identifier, synonyms, population supplementary concept word, anatomy supplementary concept word]  3) 1 or 2  4) MeSH term [Practice Guideline/ or Guideline/]  5 ("guideline*" or "practice guideline*" or "clinical practice guideline*" or recommendation* or consensus or "clinical guideline*" or criteria or "standards of care").mp. [mp=title, book title, abstract, original title, name of substance word, subject heading word, floating sub-heading word, keyword heading word, organism supplementary concept word, protocol supplementary concept word, rare disease supplementary concept word, unique identifier, synonyms, population supplementary concept word, anatomy supplementary concept word]  6) 4 or 5  7) 3 and 6  8) limit 7 to (english language and yr="2012 -Current") |
| EMBASE | 1) MeSH term [/pregnancy diabetes mellitus/]  2) MeSH term [/diabetes mellitus/]  3) ("gestational diabetes" or gdm or "gestational diabetes mellitus" or "diabetes in pregnancy").mp. [mp=title, abstract, heading word, drug trade name, original title, device manufacturer, drug manufacturer, device trade name, keyword heading word, floating subheading word, candidate term word]  4) 1 or 2 or 3  5) MeSH term [/practice guideline/]  6) ("guideline*" or "practice guideline*" or "clinical practice guideline*" or recommendation* or consensus or "clinical guideline*" or criteria or "standards of care").mp. [mp=title, abstract, heading word, drug trade name, original title, device manufacturer, drug manufacturer, device trade name, keyword heading word, floating subheading word, candidate term word]  7) 5 or 6  8) 4 and 7  9) 7 and 810)  10) limit 9 to (english language and yr = "2012 -Current") |
| CINAHL Complete | 1) “gestational diabetes" OR gdm OR "gestational diabetes mellitus" OR "diabetes in pregnancy"  2) "guideline*" OR "practice guideline*" OR "clinical practice guideline*" OR recommendation OR consensus OR standard* OR criteria  1 AND 2  Filter for: Language (English), Publication year (2012 to 2023) |
| Guideline International Network | Keyword searches:  “diabetes” “gestational diabetes” “diabetes in pregnancy”  “postnatal” “postpartum”  Filter: English |
| National Institute for Health and Clinical Excellence (NICE) | Keyword searches:  “diabetes” “gestational diabetes”  Filter: guideline |

Table S3: Conversion of certainty of evidence base of recommendations to GRADE

|  | **GRADE** | **ADA** | **NICE**  **(intervention studies)** | **NICE (diagnostic tests)** | **NHMRC** | **ACP** | **SIGN** | **USPSTF (1989)** | **Qatar** | **Turkey** |
| --- | --- | --- | --- | --- | --- | --- | --- | --- | --- | --- |
| **Certainty**  **of evidence** | High | A | 1++, 1+ | Ia, Ib | LOE I | High | 1++, 1+ | I | L1 | A |
|  | Moderate | B | 1-, 2++ | II | LOE II | Moderate | 1-, 2++ | II-1, II-2 | L2 | B |
|  | Low | C | 2+ | III | LOE III-1, | Low | 2+ | III-3 |  |  |
|  | Very low | E | 2-, 3, 4 | IV | LOE III-2, LOE III-3, LOE IV | Insufficient | 2-, 3, 4 | III | L3 | C, D |

LOE – Levels of Evidence

ACP: American College of Physicians; ADA: American Diabetes Association ; GRADE - Grading of Recommendations, Assessment, Development, and Evaluation; NGC: National Guideline Clearinghouse (NGC); NHMRC: National Health and Medical Research Council; NICE: National Institute of Care and Excellence; SIGN: Scottish Intercollegiate Guidelines Network; SORT: Strength of Recommendation Taxonomy; USPSTF: U.S. Preventive Services Task Force

Table S4: Conversion of strength of recommendations to GRADE

|  | **GRADE** | **NZ** | **SIGN** | **SORT** | **NHMRC** | **ACP** | **ACOG** | **Qatar** |
| --- | --- | --- | --- | --- | --- | --- | --- | --- |
| **Strength of Recommendation** | Strong | Strong | A, B, | A, B, C | A, B, C | Strong | A | RGA |
|  | Weak | Conditional, GPP | C, GPP | D, GPP | D, | Weak | B, C | RGB, RGC, R-GDG |

GPP: Good Practice Point, based on expert opinion / consensus

RGA: Recommendation Grade A, RGB: Recommendation Grade B, RGC: Recommendation Grade C, R-GDG: recommended based practice by Guideline Development Group

ACP: American College of Physicians; ACOG: American College of Obstetricians and Gynaecologists; NHMRC: National Health and Medical Research Council; SIGN: Scottish Intercollegiate Guidelines Network; SORT: Strength of Recommendation Taxonomy; USPSTF: U.S. Preventive Services Task Force

Table S5: Comparison of recommendations for postnatal care after GDM, strength of recommendation, certainty of evidence, and supporting data evidence rating across clinical practice guidelines.

| **Guideline title** | **Grading tool for evidence** | **Recommendation** |  | **Strength of recommendation** | **Level or quality of evidence** | **Conversion to GRADE** | **Supporting evidence rating** |
| --- | --- | --- | --- | --- | --- | --- | --- |
|  |  | **Type/ Topic** | **Description** |  |  |  |  |
| **ADA Management of Diabetes in Pregnancy: Standards of Care in Diabetes—2023** | ADA Grading System | **Postpartum glucose screening** | Screen women with a recent history of GDM at 4–12 weeks postpartum, using the 75-g OGTT and clinically appropriate nonpregnancy diagnostic criteria. | Strong | B | Moderate | MA(2) |
|  |  | **Long-term follow up** | Individuals with a history of GDM should have lifelong screening for the development of type 2 diabetes or prediabetes every 1–3 years. | Strong | B | Moderate | MA(2) |
|  |  | **Immediate postpartum care** | Insulin requirements need to be evaluated and adjusted as they are often roughly half the pre-pregnancy requirements for the initial few days postpartum. | Strong | C | Low | RC(1) |
|  |  | **Breastfeeding** | Breastfeeding is recommended to reduce the risk of maternal type 2 diabetes and should be considered when choosing whether to breastfeed or formula feed. | Strong | B | Moderate | PC(1), RC(1), R(1), MA(1) |
|  |  | **Lifestyle management** | Individuals with overweight/obesity and a history of GDM found to have prediabetes should receive intensive lifestyle interventions and/or metformin to prevent diabetes. | Strong | A | High | PC(1), MA(3) RCT(1), RC(1) |
|  |  | **Contraception** | A contraceptive plan should be discussed and implemented with all people with diabetes of reproductive potential. | Strong | A | High | CS(1), SR(1), RP(1) |
|  |  | **Psychosocial care** | Postpartum care should include psychosocial assessment and support for self-care. | Strong | E | Very low | Expert consensus |
| **Management of diabetes and its complications from preconception to the postnatal period.**  **NICE 2020** | NICE grading system | **Postpartum glucose screening** | For women who were diagnosed with gestational diabetes and whose blood glucose levels returned to normal after the birth:   - - offer a fasting plasma glucose test 6 to 13 weeks after the birth to exclude diabetes - - offer a referral into the NHS Diabetes Prevention Programme if eligible based on the results of the fasting plasma glucose test or HbA1c test. | Strong | Very low | Very low | Diagnostic accuracy of postnatal tests (13): PC(3), RC(10) Timing of postnatal tests (51): PC:(24), RC(24), CC(2), CS(1)  Referral: No evidence reported |
|  |  | **Long-term follow up** | For women having a fasting plasma glucose test as the postnatal test: they will need an annual test to check that their blood glucose levels are normal | Strong | Very low | Very low | RC(3), PC(9), |
|  |  |  | Explain to women who were diagnosed with GDM about the risks of recurrence in future pregnancies and offer them diabetes testing when planning future pregnancies | Strong | EL = 2++ | Moderate | SR(1) |
|  |  | **Immediate postpartum care** | Women who have been diagnosed with GDM should stop blood glucose‑lowering therapy immediately after birth. | Strong | Very low | Very low | Expert consensus |
|  |  |  | Before women who were diagnosed with gestational diabetes are transferred to community care, test their blood glucose to exclude persisting hyperglycaemia. | Strong | Very low (no evidence) | Very low | No evidence reported |
|  |  | **Lifestyle management** | For women who were diagnosed with gestational diabetes and whose blood glucose levels returned to normal after the birth: offer lifestyle advice (including weight control, diet, and exercise) | Strong | High | High | SR(1), RCT(2) |
| **Diagnosis and Management of Diabetes in Pregnancy. Ministry of Public Health Qatar** | Ministry of Public Health Qatar grading system | **Postpartum glucose screening** | All women should have 75g OGTT performed 4-12 weeks post-partum, Patients who missed the OGTT within the 12 weeks check should have a HBA1C performed. | Strong | Guideline development group recommendation (R-GDG) | Very low | Expert consensus |
|  |  | **Long-term follow up** | Perform annual monitoring of HBA1C or fasting blood glucose | Strong | Not reported | - | NICE 2015 |
|  |  |  | Women should be advised to maintain frequent contact with the diabetes service during the postpartum period to allow for glycaemic assessment and insulin dose adjustment. | Strong | Not reported | - | NICE 2015 |
|  |  |  | The increased risk of developing T2DM in the future should be emphasised | Strong | L1 | High | NICE 2015, ADA 2020 |
|  |  | **Immediate postpartum care** | Insulin therapy should be discontinued immediately postpartum | Strong | Not reported | - | RC(1), ADA 2020 |
|  |  | **Breastfeeding** | Women with GDM should be encouraged to breastfeed their infants as soon as possible after delivery and at frequent intervals (every 2-3 hours) and to continue for at least 3-4 months postpartum to…reduce risk of type 2 diabetes and hypertension in the mother. | Strong | Not reported | - | Other guidelines including: ADA 2020, Diabetes Canada 2018, and HKCOG 2016 |
|  |  | **Contraception** | Methods of contraception agreeable with the woman and her partner should be discussed and prescribed as appropriate | Strong | Low | Very low | NICE 2015 |
|  |  | **Lifestyle management** | It is recommended for women who were diagnosed with prediabetes to follow intensive lifestyle intervention with or without metformin to prevent diabetes | Strong | Low | Very low | ADA 2020 |
|  |  |  | Education in the postnatal period should incorporate advice on diet, physical activity and healthy weight maintenance. | Strong | Low | Very low | NICE 2015, ADA 2020 |
| **Clinical Practice Guidelines on Diabetes Mellitus in Pregnancy. Philippines 2018** | GRADE | **Postpartum glucose screening** | A 75-g OGTT is recommended at 4-12 weeks postpartum visit using nonpregnancy criteria. | Strong | High | High | SR(1), Other guidelines: ADA 2018, ACOG 2018, SOGC 2011 |
|  |  | **Long-term follow up** | If the postpartum 75-g OGTT is normal, women should be tested every 1-3 years using any glycaemic test (HbA1c, FPG or OGTT) using non-pregnancy cut-offs | Strong | High | High | MA(2), ADA 2018 |
|  |  | **Immediate postpartum care** | Women should discontinue blood glucose-lowering therapy immediately after birth | Weak | Moderate | Moderate | MA(1), ADA 2018 & 2019 |
|  |  |  | In the immediate postpartum, type 1 or 2 DM may be ruled out by fingerstick tests. FPG should be <126 mg/dL and casual plasma glucose <200 mg/dL. If DM is suspected, confirm by laboratory fasting or casual glucose tests | Strong | High | High | R(1) |
|  |  | **Breastfeeding** | Women with GDM…should be encouraged to breastfeed. | Strong | High | High | RCT(1), PC(4), RC(1), R(1) |
|  |  | **Contraception** | Nonhormonal contraceptives do not influence glycaemic control and are therefore considered first-line options in postpartum women with glucose intolerance. The copper IUD can be used safely and without any specific restrictions in women with history of GDM. | Weak | Moderate | Moderate | R(1), RP(2), WHO Guideline 2015 (Medical eligibility criteria for contraceptive use) |
|  |  | **Lifestyle management** | Adherence to a healthy lifestyle should continue in the postpartum period. Key behaviours such as healthy diet and medical nutrition therapy, regular and sufficient physical activity, and weight management should be in the centre of lifestyle modification | Strong | Not reported | Very low | R(2) |
| **Diagnosis & Management of Gestational Diabetes Mellitus: Technical and Operational Guidelines. India 2018** | No grading system reported | **Postpartum glucose screening** | Perform fasting and 2h 75g OGTT at 6 weeks postpartum to evaluate glycaemic status of woman. Cut offs for normal and abnormal blood sugar levels are: FPG: ≥ mg/dL. OGTT: Normal: <140 mg/dL, IGT: 140-199 mg/dL, Diabetes: ≥ 200 mg/dL | Strong | - | - | Not reported |
|  |  | **Long-term follow up** | If postpartum test is normal…advise women to get annual screening for diabetes in NCD clinic as per their protocols | Strong | - | - | Not reported |
|  |  |  | If postpartum test is positive/IGT, woman should be linked with NCD program for further management | Weak | - | - | Not reported |
|  |  | **Breastfeeding** | Initiate early breastfeeding…within first hour of birth | Strong | - | - | Not reported |
|  |  | **Lifestyle management** | Pregnant women with GDM are at an increased risk of Type 2 DM later in life, and should be counselled for healthy lifestyle and behaviour, particularly role of diet and exercise. | Strong | - | - | Not reported |
|  |  |  | Woman with history of GDM to be counselled about BMI and blood sugar estimation before next pregnancy | Weak | - | - | Not reported |
|  |  | **Contraception** | Counsel for postpartum family planning to potentially reduce risk of GDM in next pregnancy | Strong | - | - | Not reported |
| **Screening, Diagnosis and Management of Gestational Diabetes in New Zealand: A clinical practice guideline (2014)** | GRADE | **Postpartum glucose screening** | Remind all women with GDM and their primary care provider (at the time of hospital discharge) of the need to participate in screening at 3 months after birth and annually thereafter. | Weak | - | Low | R(2), CSS(7), RP(1), PC(2), RC(2) |
|  |  |  | Provide printed information about the importance of postpartum screening and the risk of developing type 2 diabetes. | Weak | - | High | RCT(1) |
|  |  |  | The primary care provider should offer screening for type 2 diabetes at three months postpartum using HbA1c. If the value is:  ≤ 40 mmol/mol, the result is normal. Repeat the test in one year - 41–49 mmol/mol (prediabetes or impaired fasting glucose), advise on diet and lifestyle modification. If the woman is over 35 years, a full cardiovascular risk assessment and appropriate management are indicated. Repeat test after six months - ≥ 50 mmol/mol and symptomatic ‘diabetes’, refer to medical specialist - ≥ 50 mmol/mol and asymptomatic, repeat HbA1c or FPG. | Weak | - | Very low | Expert consensus |
|  |  | **Breastfeeding** | Encourage breastfeeding and skin to skin contact as early as possible after birth (preferably within one hour). | Weak (GPP) | - | Very low | Expert consensus |
|  |  |  | Encourage and support exclusive breastfeeding for a minimum of six months. | Weak (GPP) | - | Very low | Expert consensus |
|  |  | **Immediate postpartum care** | Monitor the blood glucose before breakfast (fasting blood sugar) and two hours after meals for 24 hours after delivery. Refer to the medical team if values are between 7 mmol/L and ≥ 11 mmol/L on two consecutive occasions. If blood glucose levels are within normal range, stop monitoring after 24 hours. | Weak (GPP) | - | Very low | Expert consensus |
|  |  |  | Discontinue diabetes medication for women with a GDM diagnosis at birth. | Weak (GPP) | - | Very low | Expert consensus |
|  |  | **Contraception** | Discuss methods of contraception agreeable with the woman and her partner and prescribe contraceptives based on maternal risk factors for cardiovascular disease, in the early postnatal period | Weak (GPP) | - | Very low | Expert consensus |
|  |  | **Long term follow up** | Inform women of the increased risk of GDM in a subsequent pregnancy and the increased risk for developing type 2 diabetes. | Weak (GPP) | - | Very low | Expert consensus |
|  |  | **Lifestyle management** | Provide women diagnosed with gestational diabetes with lifestyle and dietary advice and advise on how to maintain a healthy weight. | Weak | - | High | RCT(4), SR(1) |
|  |  |  | Consider metformin in women (with previous gestational diabetes) who have HbA1c 41–49 mmol/mol and who are not successful with lifestyle modification. | Weak | - | High | RCT(1), NICE 2008 |
| **FIGO Initiative on Gestational Diabetes Mellitus: A Pragmatic Guide for Diagnosis, Management and Care 2015** | GRADE | **Postpartum glucose screening** | Glycemic status should be re-evaluated with a 75g OGTT at 6-12 weeks after delivery and interpreted based on currently recommended WHO criteria for diabetes, IFG and IGT in the non-pregnant state | Strong | Not reported | Very low | ADA 2018, Fifth International Workshop-Conference on GDM 2007 |
|  |  | **Breastfeeding** | Mothers should be encouraged and supported in initiating and maintaining breastfeeding. | Strong | Not reported | Moderate | SR(1), PC(2), RC(1), R(1) |
|  |  | **Contraception** | Women with GDM and diabetes should be encouraged to space their pregnancies to maintain and achieve optimal health between pregnancies. | Strong | Not reported | Very low | R(1), CS(1), |
|  |  | **Lifestyle management** | They should be advised to maintain a healthy lifestyle with an appropriate diet, regular exercise, and normal bodyweight to reduce the risk of future T2DM. | Strong | Not reported | High | RCT(1), PC(2), SR(1) |
| **Diabetes and Pregnancy. Diabetes Canada 2018** | Canadian Task Force on Preventive Health Care | **Postpartum glucose screening** | Women should be screened with a 75 g OGTT between 6 weeks to 6 months postpartum to detect prediabetes and diabetes. | Weak | Consensus | Very low | Expert consensus |
|  |  |  | Methods to improve postpartum testing, such as phone calls or email reminders to women should be employed to improve screening rates. | Weak | Level 3 | Low | R(1) |
|  |  | **Breastfeeding** | Women should be encouraged to continue breastfeeding for at least 3-4 months postpartum to prevent: childhood obesity;  diabetes in offspring;  reduce risk of type 2 diabetes and hypertension in the mother. | Weak | Level 3;  Level 4;  Level 3 | Low;  Very Low;  Low | MA(1)  MA(1)  MA(2), PC(2) |
|  |  | **Lifestyle management** | Women with prior GDM should receive counselling regarding healthy behaviour interventions to reduce the recurrence rate in subsequent pregnancies and reduce their increased risk of type 2 diabetes. | Weak | Level 3 | Low | PC(1), SR(1) |
|  |  |  | In women who have IGT on postpartum screening, healthy behaviour interventions with or without metformin can be used to prevent/delay the onset of diabetes. | Strong | Level 2 | Moderate | PC(1), RCT1) |
| **Diabetes and Pregnancy: An Endocrine Society Clinical Practice Guideline (2013)** | GRADE | **Postpartum glucose screening** | We recommend that a 2-hour, 75-g OGTT should be undertaken 6 to 12 weeks after delivery to rule out prediabetes or diabetes. | Strong | Moderate | Moderate | PC(1), RC (1), CS(2), R(2) |
|  |  | **Long-term follow up** | If results are normal, we recommend OGTT or other diagnostic tests for diabetes should be repeated periodically as well as before future pregnancies. | Strong | Low | Low | PC(6), RC(2), R(2) |
|  |  | **Immediate postpartum care** | We recommend that postpartum care should include measurement of fasting plasma glucose or fasting self-monitored blood glucose for 24 to 72 hours after delivery to rule out ongoing hyperglycemia. | Strong | Very low | Very low | No evidence reported |
|  |  |  | We suggest blood glucose-lowering medication should be discontinued immediately after delivery unless overt diabetes is suspected, in which case the decision to continue such medication should be made on a case-by-case basis. | Weak | Low | Low | No evidence reported |
|  |  | **Breastfeeding** | We recommend breastfeeding whenever possible. | Strong | - | Moderate | PC(4), RC(3), R(1), CS(1), CC(2) |
|  |  | **Lifestyle management** | We recommend that women receive counseling on lifestyle measures to reduce the risk of type 2 diabetes, | Strong | Very low | Very low | PC(5), CC(1) |
|  |  | **Contraception** | We recommend that the choice of a contraceptive method should not be influenced by virtue of having a history of GDM. | Strong | Moderate | Moderate | RCT(1), PC(7), RC(2), CS(1), CC(2) |
| **Screening, Diagnosis and Management of Diabetes in Pregnant Women: National Guideline, Sri Lanka (2014)** | No grading system reported | **Postpartum glucose screening** | At 6 to 8 weeks postpartum, women with GDM who became normoglycemic postpartum should be referred for screening for type 2 DM by OGTT. Fluid blood sugar is an alternative if resources are limited. Women whose fasting venous plasma glucose is above 100 mg/dl (5.5 mmol/l) must be referred for further evaluation. | Strong | - | - | Not reported |
|  |  | **Lifestyle management** | Women who screen negative at the 6-week review should receive lifestyle advice emphasising BMI control. | Strong | - | - | Not reported |
|  |  | **Long-term follow up** | Women who screen negative at the 6-week review should receive screening for noninsulin dependent diabetes mellitus annually with FBG. | Strong | - | - | Not reported |
|  |  | **Immediate postpartum care** | If mother is given insulin in the antenatal period, the dose needs adjustments to pre-pregnant doses or with diet alone in the first 48 hours after delivery. The decision should be based on her postpartum blood glucose value. If fasting blood glucose (FBG) exceeds 126 mg/dl or RBS exceeds 200 mg/dl, insulin or metformin may be required. | Strong | - | - | Not reported |
|  |  | **Contraception** | All reliable methods of family planning can be used as appropriate for the needs of the individual woman. For women with BMI >25 kg/m2, depot medroxyprogesterone acetate (DMPA) is best avoided. | Weak | - | - | Not reported |
|  |  | **Breastfeeding** | Initiate breastfeeding as  early as possible (within first half to 1 hour) to prevent hypoglycaemia in newborn | Strong | - | - | Not reported |
| **Malaysia 2017 Management of Diabetes in pregnancy** | US Preventive Services Task Force 2001 | **Postpartum glucose screening** | In women with history of gestational diabetes mellitus, OGTT should be performed at 6 weeks postpartum to detect diabetes and prediabetes. | Strong | Level III | Low | MA(1), PC(2), RC(1), Management of Type 2 Diabetes Mellitus, Ministry of Health Malaysia, 2015, NICE 2015 |
|  |  | **Long-term follow up** | If negative, annual screening should be performed. | Strong | Not reported | - | Management of Type 2 Diabetes Mellitus, Ministry of Health Malaysia, 2015 |
|  |  | **Immediate postpartum care** | Most women diagnosed with GDM should be able to discontinue their insulin immediately after delivery | Strong | Not reported | - | Management of Type 2 Diabetes Mellitus, Ministry of Health Malaysia, 2015 |
|  |  | **Breastfeeding** | Breastfeeding of at least 3 months or longer should be encouraged to reduce the risk of diabetes | Weak | Level II-2 | Moderate | PC(2), RC(1) |
|  |  | **Contraception** | There is limited data to demonstrate that Levonorgestrel-intrauterine system does not negatively affect glucose tolerance, i.e. IFG and IGT, compared with copper intrauterine device or tubal sterilisation in women with history of GDM | Weak | Level III | Low | CS(1) |
|  |  | **Lifestyle management** | In women with history of GDM, metformin and intensive lifestyle intervention during postpartum period should be considered to prevent diabetes | Strong | Level I | High | SR:RCT(1), RCT(3), NRCT(1) |
| **Guidelines for Screening, Diagnosis, and Management of Gestational Diabetes Mellitus. Iranian Endocrine Society (2020)** | The American College of Physicians’ Guideline Grading System (ACP) | **Postpartum glucose screening** | All women should be evaluated using standard 2-hour 75 g OGTT at 4 - 12 weeks after delivery. The interpretation of the results is similar to non-pregnant cases | Strong | Low | Low | R(2), RC(1) |
|  |  | **Long-term follow up** | In women with normal OGTT after delivery, the annual measurement of FPG is recommended | Strong | Low | Low | R(2), RC(1) |
|  |  | **Lifestyle management** | For all prediabetic women diagnosed after delivery, lifestyle modiﬁcations (exercise and diet), with or without metformin therapy, are recommended | Strong | High | High | PC(1), R(2) |
| **Indonesian Clinical Practice Guidelines for Diabetes in Pregnancy (2013)** | No grading system reported | **Postpartum glucose screening** | If the result (of early postpartum test) is normal, subsequent monitoring can be done at approximately 6-12 weeks post-partum by performing an OGTT. | Weak | Not reported | Very low | R(1),  Summary and recommendation of the 5th International Workshop-conference on Gestational Diabetes Mellitus 2007 |
|  |  | **Long-term follow up** | Considering that the physiological conditions of insulin resistance is expected to have fully recovered one year after giving birth, then this period is also recommended for monitoring. If the results remain normal, subsequent monitoring can be carried out every three years by doing OGTT. In the high-risk group, monitoring can be done more frequently. | Weak | Not reported | Very low | R(1),  Summary and recommendations of the fifth International Workshop-conference on Gestational Diabetes Mellitus 2007 |
|  |  | **Immediate postpartum care** | Examination of blood glucose should be routinely performed before women leave the hospital as early monitoring. Examination of fasting blood glucose ≥126 mg/dL or blood glucose 2h post prandial ≥200 mg/dL confirm the diagnosis of DM. | Strong | Not reported | Very low | R(1),  Summary and recommendations of the 5th International Workshop-conference on Gestational Diabetes Mellitus 2007 |
|  |  | **Breastfeeding** | Women are advised to breastfeed their babies. | Weak | Not reported | Very low | R(1) |
|  |  | **Contraception** | After delivery, the physician should provide advice on appropriate use of contraception. | Strong | Not reported | - | Summary and recommendations of the 5th International Workshop-conference on Gestational Diabetes Mellitus 2007 |
|  |  | **Lifestyle management** | Women are advised to undergo dietary planning with or without drugs to maintain optimal glycaemic control and adequate nutrition status. | Weak | Not reported | Very low | R(1) |
| **Diabetes Mellitus Management Guidelines. Ministry of Health, Sultanate of Oman (2015)** | No grading system reported | **Postpartum glucose screening** | All women should undergo a 2h 75-gram OGTT 6 to 12 weeks after delivery.  **A.** Diagnose diabetes, if FBG ≥7 mmol/L (126 mg/dl) and/or 2h post glucose level is ≥11.1 mmol/L (200mg/dl), **B.** Diagnose IFG, if FBG is 5.5-6.9 mmol/L (100-125 mg/dl) and **C.** Diagnose IGT if the 2h post glucose load ranges from 7.8-11 mmol/L (140-200 mg/dl) | Strong | Not reported | - | Not reported |
|  |  | **Long-term follow up** | Those with IGT should be counselled about their risk for developing overt diabetes and referred for proper management. They should have annual assessment of their glycaemic status. | Strong | Not reported | - | Not reported |
|  |  |  | Women with normal glucose tolerance should be counselled regarding their risk of developing GDM in subsequent pregnancies and type-2 diabetes in the future. Reassessment of glycaemic status should be undertaken every two years | Strong | Not reported | - | Not reported |
|  |  | **Breastfeeding** | Encourage women to breastfeed post delivery | Weak | Not reported | - | Not reported |
|  |  | **Immediate postpartum care** | A FBG done 24 hours (ideally 72 hours) post-delivery and post withdrawal of all anti hyperglycaemic agents can diagnose persistent dysglycaemia. | Weak | Not reported | - | Not reported |
|  |  | **Contraception** | Any type of contraception is acceptable. Low-dose oestrogen-progestin oral contraceptives may be used in women with a history of GDM as long as there is no medical contraindication. | Weak | Not reported | - | Not reported |
|  |  | **Lifestyle management** | Patients with IGT, IFG, or an HbA1c of 5.7–6.4% should be encouraged to lose 5–10% of their body weight and increase their physical activity to at least 150 mins/week of moderate activity. | Strong | Not reported | - | Not reported |
| **German Diabetes Association Clinical Practice Guidelines: Gestational Diabetes Mellitus (GDM), Diagnostics, Therapy and Follow-up Care 2018** | SIGN (1996) | **Postpartum glucose screening** | Perform 75g OGTT 6-12 weeks after birth. If normal, screen usually with a fasting glucose and HbA1c, if necessary OGTT every 2 years. | Strong | LoE 2+ | Low | SR(1), R(1) |
|  |  | **Long-term follow up** | After postpartum diagnosis of IFG/IGT, a yearly OGTT is recommended. | Strong | LoE 1+ | High | PC(1), SR(1) |
|  |  | **Breastfeeding** | All pregnant women with GDM should be informed about the advantages of breastfeeding for mother and child by a breastfeeding counselling service before delivery and strategies for a successful start of breastfeeding should be pointed out. | Strong | LoE 2++ | Moderate | RC(1), CS(1), PC(4) |
|  |  |  | Exclusive breastfeeding is encouraged for at least 4-6 months | Strong | LoE 2++ | Moderate | CS(1), RC(1), PC(3) |
|  |  |  | Obese pregnant women with GDM should be particularly motivated and supported to breastfeed | Strong | LoE 2+ | Low | CS(2), PC(2) |
|  |  | **Psychosocial care** | For all women after GDM, the well-being questionnaire (EPDS) should be used as a screening tool for depressive mood at the time of the OGTT 6–12 weeks after birth. | Strong | LoE 1- | Moderate | RCT(1), RC(1) |
|  |  |  | If the total score on the well-being form is > 10, a depressive mood is suspected. This suspicion should be further clarified by a specialist in order to initiate therapy in a timely manner. | Strong | EKIII | Moderate | CC(1) |
|  |  | **Lifestyle management** | Women with glucose tolerance disorder after GDM should receive detailed advice and instructions on intervention measures (lifestyle modification). Which includes: • needs-adapted nutrition; • Weight normalization; • physical activity; • Smoking cessation. | Strong | LoE 1++ | High | RCT(1), PC(1), R(1) |
| **Clinical Practice Guidelines: Diabetes Mellitus. Ministry of Health Singapore (2014)** | GRADE | **Postpartum glucose screening** | For women with GDM, a 75 g 2h OGTT should be performed 6–12 weeks postpartum and the woman reclassified and counselled according to criteria accepted in the non-pregnant state. | Weak | Level 2+ | Low | ADA 2013 |
|  |  | **Long-term follow up** | Women should have lifelong screening for the development of prediabetes or diabetes at least once every 3 years. | Weak | Level 4 | Very low | SIGN 2010 |
|  |  | **Immediate postpartum care** | Women should discontinue glucose- lowering treatment immediately after birth and their blood glucose levels monitored. | Weak | Level 4 | Very low | SIGN 2010 |
|  |  | **Breastfeeding** | Breastfeeding is recommended for infants of women with GDM | Weak | Level 3 | Low | PC(2), CS(1) |
|  |  | **Lifestyle management** | Women should be offered lifestyle advice aimed at diet modification, weight control and increasing physical activity to reduce their risk of subsequent development of diabetes. | Weak | Level 4 | Very low | R(1), NICE 2008 |
|  |  | **Contraception** | Low-dose oestrogen-progestin oral contraceptives and intrauterine devices are not contraindicated in women with previous GDM. | Weak | Level 3 | Low | R(1) |
| **Clinical practice guidelines on diabetes mellitus and pregnancy: ΙI. Gestational diabetes mellitus. Greece 2020** | No grading system reported | **Postpartum glucose screening** | It is recommended to perform an OGTT 2-3 months postpartum for all women with GDM. | Strong | Not reported | - | Not reported |
|  |  | **Long-term follow up** | If the OGTT results are within the reference range, an OGTT should be repeated every 1–3years, considering other risk factors | Strong | Not reported | - | Not reported |
|  |  | **Immediate postpartum care** | It is not recommended to routinely measure blood glucose concentrations after delivery in women with GDM | Strong | Not reported | - | Not reported |
|  |  |  | It is recommended to quit insulin therapy after delivery for women with GDM treated with insulin. | Strong | Not reported | - | Not reported |
|  |  | **Breastfeeding** | Breastfeeding is recommended and should be encouraged in women, especially when obese. | Strong | Not reported | - | Not reported |
|  |  | **Lifestyle management** | It is recommended that the woman adopt a healthy lifestyle with the preservation of ideal body weight, balanced diet, and physical activity. | Strong | Not reported | - | Not reported |
|  |  | **Contraception** | Hormonal contraception with progestogen-only pills is not recommended. Low-dose oestrogen-progestogen combined oral contraception pills are not contraindicated, as they do not increase the risk of diabetes mellitus. The use of intrauterine devices with progestogen (Mirena®) or copper is not contraindicated. | Strong | Not reported | - | Not reported |
| **Management of Diabetes: A National Clinical Guideline 2017. Scottish Intercollegiate Network Guideline (SIGN)** | SIGN (1996) | **Postpartum glucose screening** | Where diabetes is not apparent immediately after delivery, glucose tolerance should be reassessed at least 6 weeks postpartum with a minimum of fasting glucose and with 75g OGTT if clinically indicated. | Weak | GPP | Very low | Expert consensus |
|  |  | **Long-term follow up** | An annual assessment of glycaemia using fasting glucose or HbA1c should be carried out | Weak | GPP | Very low | Expert consensus |
|  |  |  | Women who have developed GDM should be reminded of the need for pre-conception counselling and appropriate testing to detect progression to type 2 diabetes. | Weak (C) | GPP | Very low | Expert consensus |
|  |  | **Breastfeeding** | Breast feeding should be encouraged to benefit mother and baby, but it may necessitate insulin dose adjustment and a dietetic review. | Weak | GPP | Very low | Expert consensus |
|  |  | **Contraception** | Appropriate contraception should be provided, and the importance of good glycaemic control emphasised. | Weak | GPP | Very low | Expert consensus |
|  |  | **Lifestyle management** | Women who have developed GDM should be given diet, weight control and exercise advice. | Weak [C] | Level 2++ | Moderate | SR(1), R(1), RCT(1) |
| **Management of Diabetes: A handbook for general practice. RACGP 2020** | NHMRC levels of evidence and grades of recommendation (2009–16) | **Postpartum glucose screening** | Postnatal education and support are important in preventing or delaying the onset of diabetes in the future, and women should be encouraged to attend postnatal testing | Strong | Consensus | Very low | NHMRC 2019 - National evidence-based guideline for case detection and diagnosis of type 2 diabetes. |
|  |  |  | Women should have a 75 g 2h OGTT, preferably at 6–12 weeks postpartum, with classification according to World Health Organization criteria. | Strong | Consensus | Very low | ADIPS 2014 Consensus Guidelines |
|  |  | **Long-term follow up** | If results are normal, conduct a FBG and HbA1c test every 3 years. | Strong | C | Low | NHMRC 2009 |
|  |  |  | Women contemplating another pregnancy should have an OGTT annually | Strong | C | Low | ADIPS 2014 Consensus Guidelines |
|  |  | **Lifestyle management** | Women with IGT/IFG should be referred to lifestyle intervention programs to:  • achieve and maintain a 7% reduction in weight  • increase moderate-intensity physical activity to at least 150 minutes per week | Strong | A | High | ADA 2019 |
|  |  | **Breastfeeding** | In women with GDM, discuss the benefits of breastfeeding in reducing the risk of the woman developing type 2 diabetes in the future | Weak | Not reported | - | Department of Health. Clinical practice guidelines: Pregnancy care. 2019. |
| **SEMDSA 2017 Guidelines for the Management of Type 2 diabetes mellitus** | Strength of Recommendations Taxonomy for grading of recommendations (SORT) 2004 | **Postpartum glucose screening** | All patients with hyperglycaemia in pregnancy and normoglycaemia post-delivery should be reassessed with a 2-hour OGTT at 6 weeks postpartum. | Strong | Grade A | High | Not reported |
|  |  | **Long-term follow up** | Annual screening for diabetes should be performed if the result is normal with HbA1C test | Strong | Grade B | Moderate | Not reported |
|  |  |  | Persons with IFG/IGT must be screened for other cardiovascular risk factors, including metabolic syndrome. These risk factors must be managed optimally. | Strong | Grade A | High | Not reported |
|  |  | **Breastfeeding** | Breastfeeding should be encouraged wherever possible. | Strong | Not reported | - | Not reported |
|  |  | **Contraception** | Contraception should be discussed and implemented. Most forms of contraception are safe and effective in women with diabetes. | Strong | Not reported | - | Not reported |
|  |  | **Lifestyle management** | Consider metformin for individuals who have deteriorating FPG or 2-h postprandial after 6 months, who may or may not have participated in an intensive lifestyle intervention programme. | Strong | Grade A | High | RCT(1) |
|  |  |  | Offer intensive lifestyle interventions as part of a structured programme that is based on proven principles. Use this to support the individual with IGT/IFG to: 1. Achieve and maintain weight loss >5%  2. Modify dietary patterns focusing on  a. Reducing energy from fat to ≤30%  b. Reducing energy from saturated fat to ≤10%  c. Increasing fibre intake ≥15 g/1,000 kcal  3. Increase moderate intensity physical activity ≥ 150 minutes per week | Strong | Grade A | High | RCT(1), SR(1) |
| **Clinical Practice Guideline for Diagnosis, Treatment and Follow-up of Diabetes Mellitus and Its Complications. Turkey 2019** | Society of Endocrinology and Metabolism of Turkey (SEMT) grading system | **Postpartum glucose screening** | Postpartum blood glucose screening should be performed with 75-g OGTT between 4-12 weeks postpartum even if the values are back to normal range (A1c is also recommended for suspected cases) | Strong | D | Very low | Expert consensus |
|  |  | **Long-term follow up** | If normal, screening should be repeated every 3 years | Strong | D | Very low | Expert consensus |
|  |  | **Breastfeeding** | Breastfeeding should be started as soon as possible after the birth, and should be encouraged for at least 6 months | Strong | D | Very low | Expert consensus |
|  |  | **Lifestyle management** | Nutrition counselling should be provided during the pregnancy for women diagnosed with GDM and continued in the postpartum period | Strong | D | Very low | Expert consensus |
|  |  |  | Individuals with GDM history and HbA1C >5.7% (39 mmol/mol) should be included in lifestyle modification programs (healthy nutrition, ensuring weight loss if necessary and increasing the physical activity level to reduce risk of type 2 diabetes. | Strong | Not reported | - | Not reported |
| **ACOG Practice Bulletin No. 180, 2017** | U.S. Preventive Services Task Force | **Postpartum glucose screening** | Screening at 4–12 weeks postpartum is recommended for all women who had GDM to identify women with diabetes, IFG or IGT. | Strong | Level C (expert opinion) | Very low | ADA 2017 |
|  |  | **Lifestyle management** | Women with IFG/IGT, or diabetes should be referred for preventive or medical (metformin) therapy. Offer MNT, and weight loss and physical activity counselling as needed. | Strong | Level C (expert opinion) | Very low | ADA 2017 |
|  |  | **Long-term follow up** | The ADA and ACOG recommend repeat testing every 1–3 years for women who had normal postpartum screening test results. In the presence of IFG or IGT, offer a yearly glycaemic assessment. | Strong | Level C (expert opinion) | Very low | ADA 2017 |
| **GDM: South Asian Federation of Endocrine Societies Recommendation and Action Plan (2018)** | No grading system reported | **Postpartum glucose screening** | Women with GDM should be screened for diabetes 6 weeks postpartum (linked to child immunisation) with 75g 2hour OGTT using non-pregnant OGTT criteria. | Strong | Not reported | - | Not reported |
|  |  | **Long-term follow up** | If blood glucose is normal, re-assessment should be done annually with 75g 2hour OGTT or HbA1C. If prediabetes, re-assessment should be done 6 monthly. | Strong | Not reported | - | Not reported |
|  |  |  | Screening for all components of metabolic syndrome should be offered. | Strong | Not reported | - | Not reported |
|  |  |  | All mothers with history of gestational diabetes should be counselled about screening for GDM in subsequent pregnancy | Strong | Not reported | - | Not reported |
|  |  | **Immediate postpartum care** | After delivery at least 1 fasting and 1 post meal blood glucose before discharge should be measured in GDM patients who were managed by MNT and FBG and post meals BG should be monitored for at least 24 hours who were managed with insulin. Possibility of type 2 diabetes should be considered. If immediate post-delivery (i.e. 1-3 days) blood glucose is suggestive of DM, then should be confirmed by FBG (>7 mmol/l) or post-prandial glucose (>11.1mmol/l) | Strong | Not reported | - | Not reported |
|  |  |  | Women who were on metformin can stop the medication. Those who were on low dose insulin (<0.5units/kg/day) can stop insulin and glucose levels monitored. | Weak | Not reported | - | Not reported |
|  |  | **Breastfeeding** | Exclusive breast feeding is recommended as this reduces mother's and offspring's obesity and prevent development of future type 2 DM of mother and reduces the risk of neonatal hypoglycaemia in offspring | Strong | Not reported | - | Not reported |
|  |  | **Contraception** | Low dose oestrogen-progesterone can be offered for contraception. Medroxyprogesterone preparations can increase risk of vascular complications with long-term use. However, long acting levonorgestrel based systems are relatively safe. Barrier method and non-hormonal intrauterine device can be used safely in all women. | Weak | Not reported | - | Not reported |
|  |  | **Lifestyle management** | If the pregnancy has motivated the adoption of a healthier diet, building on these gains to support weight loss is recommended in the postpartum period. | Strong | Not reported | - | Not reported |
|  |  |  | Women whose test results show prediabetes should be put on either MNT alone or MNT and metformin. | Strong | Not reported | - | Not reported |
|  |  | **Psychosocial care** | Special attention should be paid to the psychological well-being of women with GDM who experience fetal loss - refer to a mental health professional as and when needed. | Strong | Not reported | - | Not reported |
| **Standards of Polish Society of Gynecologists & Obstetricians in management of women with diabetes (2018)** | No grading system reported | **Postpartum glucose screening** | It is recommended to perform a 75g OGTT 6 weeks after delivery with the results interpreted according to WHO guidelines for the general population. | Strong | Not reported | - | Guidelines on the management of diabetic patients. A position of Diabetes Poland. 2018 |
|  |  | **Long-term follow up** | In case of negative OGTT results, the test should be repeated annually. | Strong | Not reported | - | Not reported |
|  |  | **Immediate postpartum care** | In cases of GDM treated with diet alone, the patient should be encouraged to self-monitor her glucose levels while fasting and 2h postprandial for a few days after childbirth. If blood glucose levels are within normal range, glycemic control can be discontinued. | Strong | Not reported | - | Not reported |
|  |  |  | In cases of GDM treated with insulin, therapy should be discontinued right after delivery while glucose monitoring should be performed when fasting and 2h postprandial. | Strong | Not reported | - | Not reported |
|  |  | **Breastfeeding** | Patients with GDM should be encouraged and motivated to breastfeed for a minimum of 6 months. | Strong | Not reported | - | Not reported |
| **Clinical Practice Guidelines for Diabetes Management in Nigeria (2013)** | No grading system reported | **Postpartum glucose screening** | Women with GDM should be screened for diabetes 6–12 weeks postpartum and should be reclassified and followed up with subsequent screening for the development of diabetes or pre-diabetes (WHO criteria) | Strong | Not reported | - | Not reported |
|  |  | **Breastfeeding** | Promote and support breastfeeding for 6 months from birth to reduce risk of undernutrition, promote infant growth and development, and reduce risk of obesity and NCDs later in life | Weak | Not reported | - | Not reported |
|  |  | **Lifestyle management** | Promote healthy diet and physical activity through education to prevent development of type 2 diabetes | Weak | Not reported | - | Not reported |
| **HKCOG Guidelines for the Management of Gestational Diabetes Mellitus (2016)** | No grading system reported | **Postpartum glucose screening** | Women should be offered a postnatal glucose screening test at 6-12 weeks to exclude DM. | Strong | Not reported | - | Not reported |
|  |  |  | This testing can be done with either OGTT or HbA1c (with or without FBG). | Weak | Not reported | - | Not reported |
|  |  | **Long-term follow up** | Women with higher risk for progression based on test result or existing risk factors … should require more frequent testing (yearly) than those at lower risk (3-yearly). | Strong | Not reported | - | Not reported |
|  |  | **Breastfeeding** | Women should be encouraged to breastfeed. | Strong | Not reported | - | Not reported |
|  |  | **Lifestyle management** | Women should be informed about the increased risk of future DM and hyperglycaemia in future pregnancy and should be offered lifestyle advice including weight control, diet, and exercise. | Strong | Not reported | - | Not reported |

ADA **-** American Diabetes Association; ADIPS - Australian Diabetes in Pregnancy Society; AHCPR – Agency for Health Care Policy and Research; FIGO – International Federation of Gynecology and Obstetrics; GRADE - Grading of Recommendations, Assessment, Development, and Evaluation; HKCOG – Hong Kong College of Obstetricians and Gynaecologists; NHMRC - National Health and Medical Research Council; SOGC **-** Society of Obstetricians and Gynaecologists of Canada; SIGN - Scottish Intercollegiate Guidelines Network; RACGP - The Royal Australian College of General Practitioners; WHO – World Health Organization

BMI – body mass index; DM – diabetes mellitus; EPDS – Edinburgh Postnatal Depression Scale; FBG – fasting blood glucose; FPG – Fasting Plasma Glucose; GDM – gestational diabetes mellitus; GPP – Good Practice Points; HbA1c – Haemoglobin A1c; IFG - impaired fasting glucose; IGT - impaired glucose tolerance; LoE – Level of Evidence; MNT – Medical Nutrition Therapy; NCD – Non-communicable disease; OGTT – oral glucose tolerance test

CC – case-control; CS – cross-sectional study; MA _ meta-analyses; NRCT – non-randomized control trials; PC – prospective cohort; R – review; RC – retrospective cohort; RCT – randomized control trial; SR – systematic review; SR:RCT – systematic review of randomized controlled trials;
